# Reaction Behavior of [1,3-Diethyl-4,5-diphenyl-1*H*-imidazol-2-ylidene] Containing Gold(I/III) Complexes against Ingredients of the Cell Culture Medium and the Meaning on the Potential Use for Cancer Eradication Therapy

**DOI:** 10.1021/acs.jmedchem.3c00589

**Published:** 2023-06-09

**Authors:** Paul Kapitza, Amelie Scherfler, Stefan Salcher, Sieghart Sopper, Monika Cziferszky, Klaus Wurst, Ronald Gust

**Affiliations:** † Department of Pharmaceutical Chemistry, Institute of Pharmacy, Center for Molecular Bioscience Innsbruck, University of Innsbruck, Innrain 80/82, Innsbruck A-6020, Austria; ‡ Department of Internal Medicine V, Haematology & Oncology, Medical University Innsbruck, Anichstrasse 35, Innsbruck A-6020, Austria; § Department of General, Inorganic and Theoretical Chemistry, University of Innsbruck, Innrain 80/82, Innsbruck A-6020, Austria

## Abstract

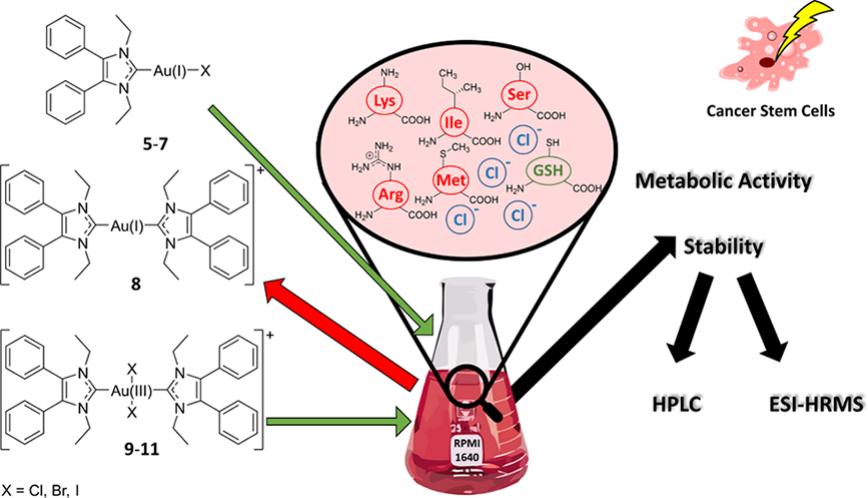

The reactivities of halido[1,3-diethyl-4,5-diphenyl-1*H*-imidazol-2-ylidene]gold(I) (chlorido (**5**), bromido (**6**), iodido (**7**)), bis[1,3-diethyl-4,5-diphenyl-1*H*-imidazol-2-ylidene]gold(I) (**8**), and bis[1,3-diethyl-4,5-diphenyl-1*H*-imidazol-2-ylidene]dihalidogold(III) (chlorido (**9**), bromido (**10**), iodido (**11**)) complexes against ingredients of the cell culture medium were analyzed by HPLC. The degradation in the RPMI 1640 medium was studied, too. Complex **6** quantitatively reacted with chloride to **5**, while **7** showed additionally ligand scrambling to **8**. Interactions with non-thiol containing amino acids could not be detected. However, glutathione (GSH) reacted immediately with **5** and **6** yielding the (NHC)gold(I)-GSH complex **12**. The most active complex **8** was stable under *in vitro* conditions and strongly participated on the biological effects of **7**. The gold(III) species **9**–**11** were completely reduced by GSH to **8** and are prodrugs. All complexes were tested for inhibitory effects in Cisplatin-resistant cells, as well as against cancer stem cell-enriched cell lines and showed excellent activity. Such compounds are of utmost interest for the therapy of drug-resistant tumors.

## Introduction

The need for new and improved drugs in tumor therapy is growing rapidly, because of the occurrence of an increased number of tumoral diseases that are resistant to conventional anticancer drugs. Especially, metallodrugs are promising candidates to circumvent acquired and intrinsic resistance.^1−6^ In particular, gold complexes were established as effective cytostatics, since the approval of Auranofin in 1985 as a drug against rheumatoid arthritis.^7−11^ Auranofin is now approved by the FDA for phase-II clinical trials in cancer therapy.^12^

**Chart 1:**
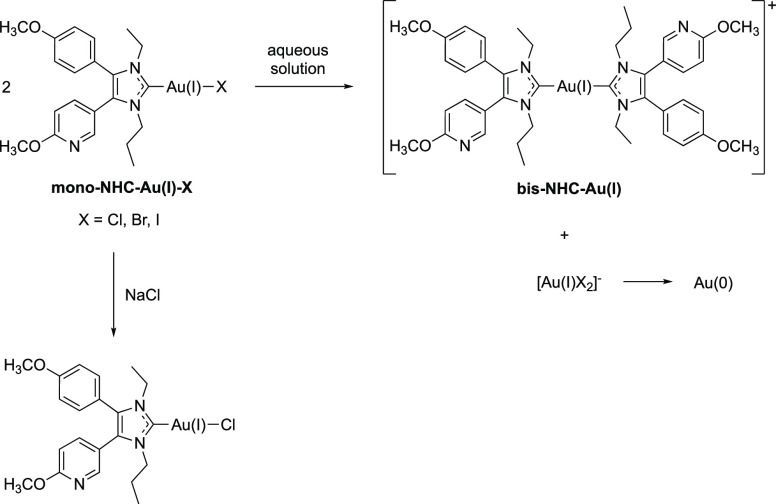
Reaction Behavior of Halido[3-ethyl-4-(4-methoxyphenyl)-5-(2-methoxypyridin-5-yl)-1-propyl-1*H*-imidazol-2-ylidene]gold(I) Complexes (mono-NHC-Au(I)-X, X = Cl, Br, I)

During the past years, gold containing drug candidates with *N*-heterocyclic carbenes (NHC) as ligands received high attention, because of their easy synthesis and good biological activity.^8,13−16^ Antiproliferative effects of (NHC)gold(I)-X complexes where X represents a leaving group are well documented. This particularly applies to halido derivatives. While the meaning of halido leaving groups at platinum(II) complexes on the reactivity in water and thus also on the cytotoxicity has been intensively studied,^17,18^ comparable investigations with (NHC)gold(I)-X (X = Cl, Br, I) complexes are rather rare. In aqueous solution, nucleophiles can force ligand exchange reactions, but ligand scrambling is observed, too.^19^ Chloride, present in cell culture media, transforms the bromido complex to the chlorido species as demonstrated in time-dependent experiments on the example of halido[3-ethyl-4-(4-methoxyphenyl)-5-(2-methoxypyridin-5-yl)-1-propyl-1*H*-imidazol-2-ylidene]gold(I) complexes (mono-NHC-Au(I)-X, Chart 1). Kinetic reactions with other nucleophiles of the media, e.g., amino acids or glutathione (GSH), were not investigated yet, although such reaction is described for other metal complexes, including Cisplatin.^20−22^ However, even for Cisplatin, this issue has not yet been fully elucidated as recently discussed by Hall.^23^

Unlike platinum(II) complexes, aquation is not the preferred reaction of mono-NHC-Au(I)-X complexes in water. Instead, they show partially ligand scrambling resulting in the cationic [(NHC)_2_Au(I)]^+^ complex (bis-NHC-Au(I), Chart 1) and [Au(I)X_2_]^−^ ion. The latter finally decomposes to Au(0).^24,25^

Especially, the mono-NHC-Au(I)-I complex displayed this reaction during the first minutes of incubation.

Such transformations are of high importance for the interpretation of the biological results, because there are several clues that the antiproliferative activity of [(NHC)_2_Au(I)]^+^ species is generally more than 5-fold higher than that of the related (NHC)gold(I)-X (X = Cl, Br, I) complexes.^26−31^

In water-free organic solvent, e.g., dimethylformamide (DMF), the mono-NHC-Au(I)-X complexes (Chart 1) were stable for 72 h (degradation <2%),^24^ so stock solutions for the *in vitro* testing can be prepared. The final concentrations for the cell culture experiments were achieved by dilution with the respective medium, to realize a maximum amount of organic solvent of 0.1%.

It is assumed that the first contact with the medium started the ligand scrambling of mono-NHC-Au(I)-X and present nucleophiles force the degradation further. Therefore, we investigated in a preliminary study the stability of the complexes in acetonitrile/water = 50/50 (v/v) in the presence of 0.9% NaCl. To simulate the reaction with medium ingredients, the complexes were treated with a 20-fold excess of iodide as a model nucleophile.

Mono-NHC-Au(I)-Br and mono-NHC-Au(I)-I rapidly reacted in NaCl solution to mono-NHC-Au(I)-Cl, but also underwent a distinct degradation to bis-NHC-Au(I) (Chart 1).^19,27,32^

Iodide as the nucleophile led to a fast reaction to mono-NHC-Au(I)-I and subsequently to an increased formation of bis-NHC-Au(I).

These interesting results induced us to monitor the transformation of (NHC)gold(I) complexes in the cell culture medium more detailed on the example of halido[1,3-diethyl-4,5-diphenyl-1*H*-imidazol-2-ylidene]gold(I) (chlorido (**5**), bromido (**6**), iodido (**7**)), bis[1,3-diethyl-4,5-diphenyl-1*H*-imidazol-2-ylidene]gold(I) (**8**), and bis[1,3-diethyl-4,5-diphenyl-1*H*-imidazol-2-ylidene]dihalidogold(III) (chlorido (**9**), bromido (**10**), iodido (**11**)) complexes.

It is very likely that reaction products reduce on the one hand the concentration of the investigated complex and on the other hand have antiproliferative activity themselves.

The RPMI 1640 cell culture medium used in this study contains, besides numerous l-amino acids, vitamins such as biotin, i-inositol, or vitamin B12. Also present is a large amount of glucose and chloride containing inorganic salts (sodium and potassium chloride) as well as the tripeptide GSH.

In this study, we reacted the halido(NHC)gold(I) complexes **5**–**7** with main ingredients of the medium, e.g., chloride and amino acids. The products, in particular, the [(NHC)_2_Au(I)]^+^ species, were time-dependent quantified by high-pressure liquid chromatography (HPLC) analysis.

Besides the reaction with each nucleophile, the degradation of the complexes in a complete RPMI 1640 cell culture medium was monitored.

Another relevant aspect is the presence of GSH in the cell culture medium. The glutathione ″supersystem″ causes detoxification due to binding of GSH to heavy metals and other toxins.^33−35^ Since it is well known that gold complexes have a high affinity for thiol-bearing compounds,^36^ we also quantified the reaction of the (NHC)gold complexes **5**–**7** with GSH.

Of further interest is the redox behavior of GSH, which allows a reduction of Au(III) to Au(I). Therefore, we included in this study [(NHC)_2_Au(III)X_2_]^+^ complexes with X = Cl (**9**), Br (**10**), and I (**11**) and determined their stability in the presence of GSH.

Furthermore, the complexes **5**–**11** were investigated for antiproliferative effects in various cell lines, sensitive and resistant to known cytostatics, as well as in cancer cell lines with enriched proportions of cells with stem cell characteristics (cancer stem cells, CSC).

## Results and Discussion

### Synthesis and Structural Characterization

**Scheme 1:**
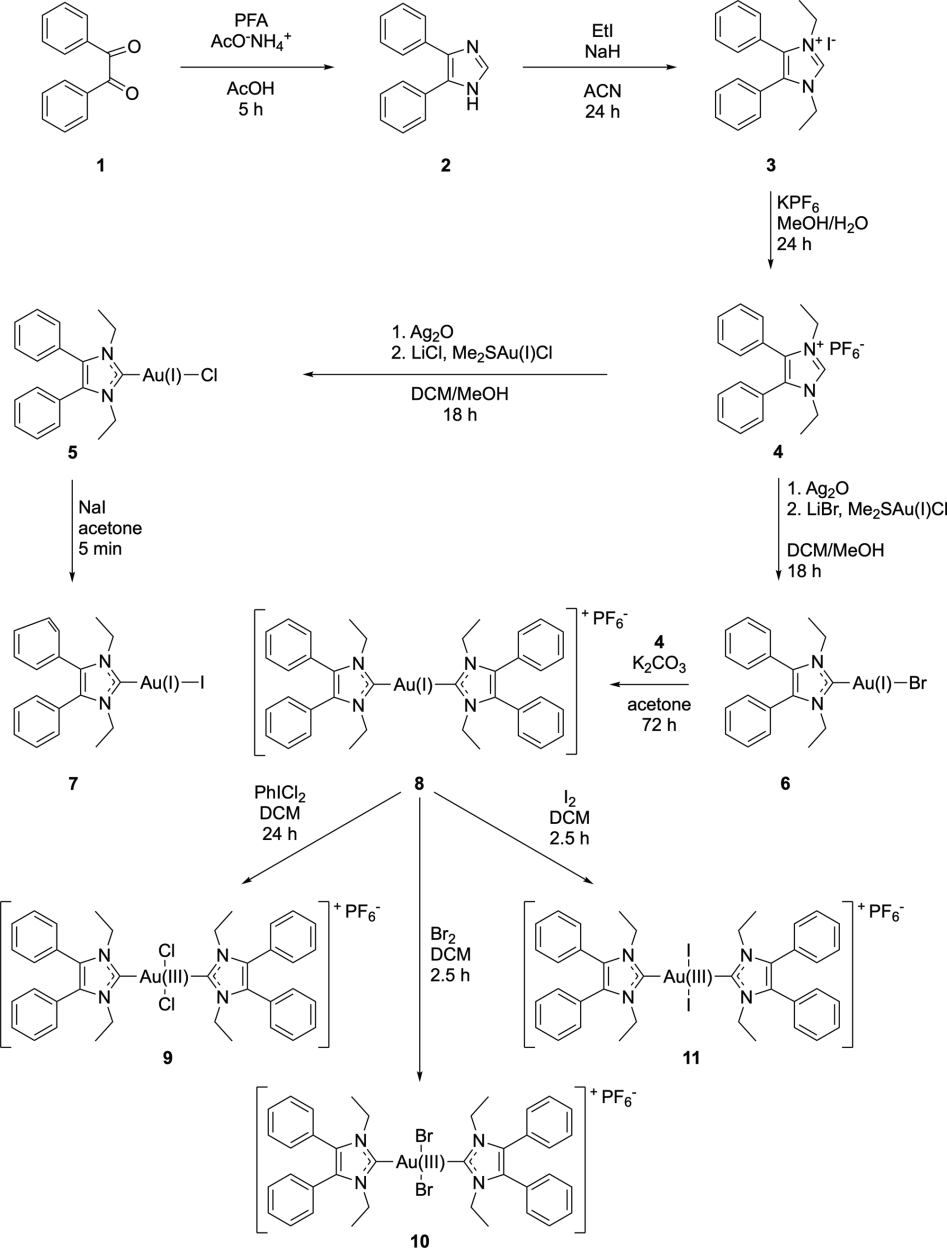
Synthetic Routes to Obtain Complexes **5**–**11**

The synthesis of the complexes **5**–**11** was performed in a multistep procedure as depicted in Scheme 1.

The reaction of benzil (**1**) with paraformaldehyde (PFA) and ammonium acetate in concentrated acetic acid yielded the 4,5-diphenyl-1*H*-imidazole **2**,^37^ which was subsequently *N*,*N*′-diethylated with iodoethane and sodium hydride (→**3**) in anhydrous acetonitrile (ACN).^32,38^

Since iodide is a strong coordinative anion, which forces the formation of iodido(NHC)gold(I) complexes as a by-product in the synthesis of the chlorido/bromido(NHC)gold(I) derivatives,^19^ an anion exchange with **3** was carried out using potassium hexafluorophosphate in methanol (MeOH)/water (→**4**).

The chlorido(NHC)gold(I) complex **5** and bromido(NHC)gold(I) complex **6** were synthesized from the 1,3-diethyl-4,5-diphenyl-1*H*-imidazolium hexafluorophosphate salt (**4**) with silver oxide, chlorido(dimethyl)sulfidegold(I), and a 5-fold excess of lithium chloride or lithium bromide, respectively, following the procedure as previously described.^19,27,38,39^ The complexes were purified by silica column chromatography with dichloromethane (DCM) and MeOH.^40^

A simple Cl/I exchange reaction of **5** with sodium iodide in dry acetone for only 5–10 min at room temperature (rt) provided the iodido(NHC)gold(I) complex **7** in high purity.^19^

In a similar way, the [(NHC)_2_Au(I)]^+^ complex **8** was prepared from the bromido(NHC)gold(I) complex **6** by reaction with an equimolar amount of **4** for several days.^11,26,27^ To separate **8** from unreacted educts, purification by column chromatography was necessary.^11,27^

Oxidation of **8** with either dichloroiodobenzene (PhICl_2_), bromine, or iodine yielded the (NHC)gold(III) derivatives **9**–**11**, respectively.^26,41^ All compounds could be obtained in high purity as determined by HPLC (>97%, Figures S1–S7). For structural characterization, nuclear magnetic resonance (NMR) spectroscopy and high-resolution mass spectrometry (HR-MS) were used.

The influence of the halido ligands on the NHC resonances in the ^1^H NMR spectra of **5**–**11** (Figures S8–S14) was only marginal and did not allow a structural discrimination. However, this is possible based on the resonances of the C2 carbon directly bound to gold(I/III). From the ^13^C NMR spectra (Figures S15–S21), it is obvious that the chemical shift depends on the bound halide and oxidation state of the metal center.

As expected, a low-field shift dependent on the electronic density was observed in the (NHC)Au(I)-X series (**5** (X = Cl: 169.21 ppm) < **6** (X = Br: 172.83 ppm) < **7** (X = I: 179.96 ppm)).^31,40,42^

This resonance was located in case of the [(NHC)_2_Au(I)]^+^ complex **8** at 182.63 ppm. Oxidation to [(NHC)_2_Au(I)X_2_]^+^ caused a strong high-field shift: **11** (X = I: 142.84 ppm) < **10** (X = Br: 149.99 ppm) < **9** (X = Cl: 153.26 ppm).

**Figure 1:**
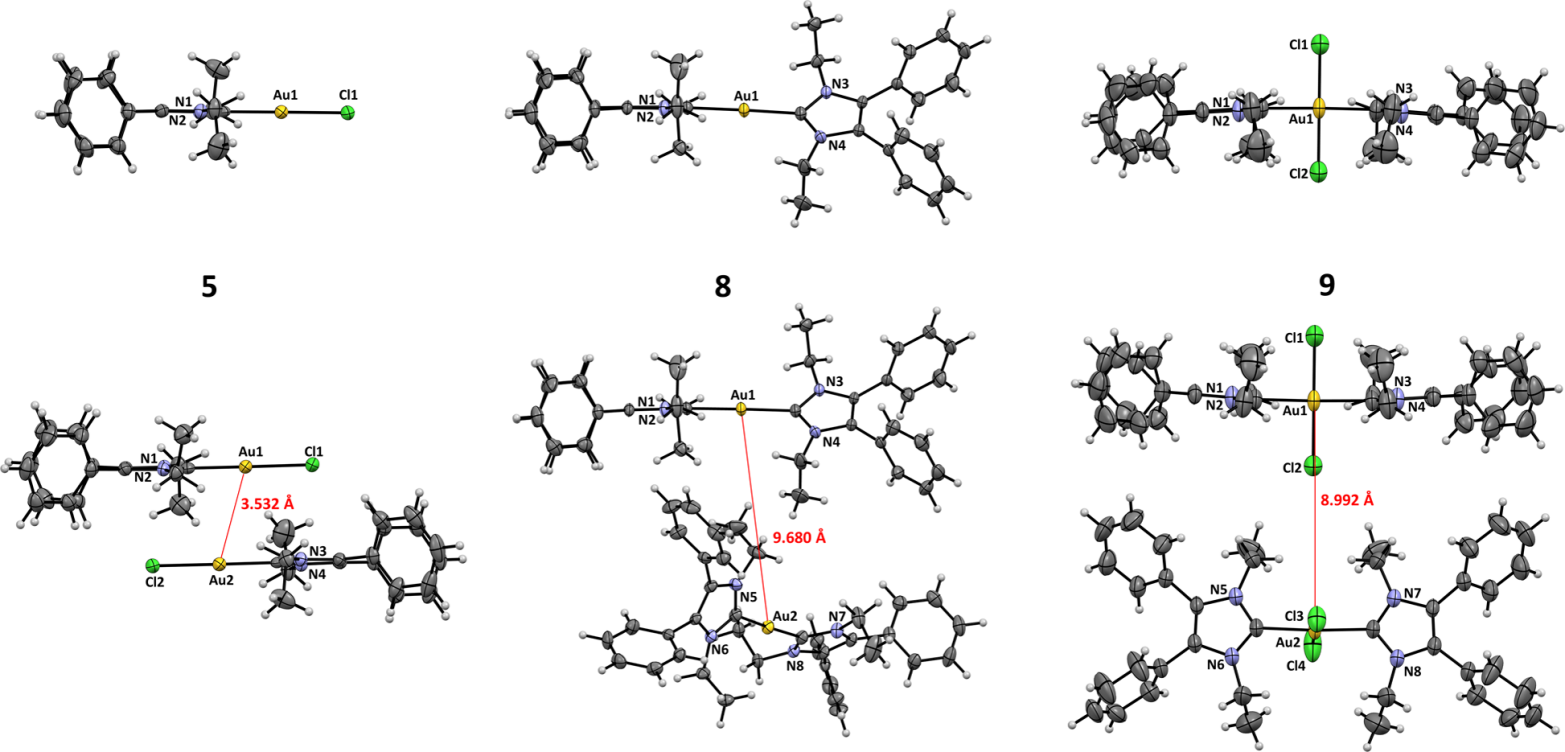
ORTEP of **5**, **8**, and **9**. For **6**, **7**, **10**, and **11** see the Supporting Information.

X-Ray crystallography confirmed the structure of the complexes **5**–**11** (Figure 1 and Figures S22–S25). Crystals were grown by slow crystallization over several days from an ACN solution (for crystallographic data, see Tables S1–S8).

The spatial structures of the NHC ligand and the length of the NHC-gold bond (1.995–2.028 Å) were nearly identical in all (NHC)Au(I)-X complexes, while the Au(I)-X distance (2.299 Å (**5**), 2.394 Å (**6**), and 2.584 Å (**7**)) increased with the atomic radius of the halide. The complexes formed slightly distorted columnar structures with Au(I)-Au(I) distances of 3.532 and 3.579 Å in **5** and **6**, as well as of 4.011 Å in **7**. These values point to weak aurophilic interactions.

The [(NHC)_2_Au(I)]^+^ complex **8** (Au(I)-Au(I) distance: 9.680 Å) and [(NHC)_2_Au(III)X_2_]^+^ complexes **9**–**11** (Au(I)-Au(I) distances: 8.992 Å (**9**), 11.898 Å (**10**), and 7.921 Å (**11**)) showed no Au(I)-Au(I) interactions in the crystal.

The ligands took an orientation, in which the NHC moieties are perpendicularly arranged.

Oxidation to Au(III) and coordination of two halido ligands resulted in a square planar environment at the metal center. The NHCs were aligned perpendicular to this plane due to the steric repulsion with the halido ligands.

### Reactivity Studies

In a previous study, we established an HPLC system, which allows the quantification of mono-NHC-Au(I)-X (X = Cl, Br, and I) in the presence of nucleophiles. This method was adapted to the analysis of the complexes **5**–**11**.

As mentioned above, the RPMI 1640 medium contains as nucleophiles besides chloride a variety of amino acids and GSH, which can coordinate to the metal or be involved in the redox reaction with gold(I/III).

All nucleophiles (chloride, amino acids, and GSH) were separately reacted with **5**–**11** in ACN/water = 50/50 (v/v) mixtures at a complex concentration 0.5 mM. This solvent composition was chosen to prevent precipitation of the complex and the formed degradation products.

As a source for chloride, a NaCl (12.0 g/L)/KCl (0.8 g/L) solution was prepared. After mixing with ACN (50/50 (v/v)), the concentration corresponds to that in the medium.

Amino acids, as well as GSH, were used in 20-fold excess, as realized at a complex concentration of 10 μM in the cell culture medium. Thereto, the respective complex, dissolved in ACN (1 mM), was combined with the same amount of aqueous solution of the nucleophile (20 mM).

The mixture was then incubated for 24 h at rt and monitored at various time points via HPLC using an RP-C18 column and gradient elution (70/30 (v/v) to 90/10 (v/v)) of ACN/water (0.1% trifluoroacetic acid (TFA)).

It must be mentioned that ACN can act as a weak nucleophile and is able to displace the halide from the gold(I) center of (NHC)Au(I)-X complexes, resulting in the ((NHC)Au(I)-ACN) species. This reaction product would be the same for **5**–**7**.

However, the chromatograms of the complexes in ACN solution (Figures S1–S3) show peaks with different retention times (**5**: *t*_R_ = 5.13 min, **6**: *t*_R_ = 5.92 min, and **7**: *t*_R_ = 7.00 min). An additional peak probably caused by (NHC)Au(I)-ACN is not present. Transformation to the Au(I)-ACN can therefore be excluded.

**Figure 2:**
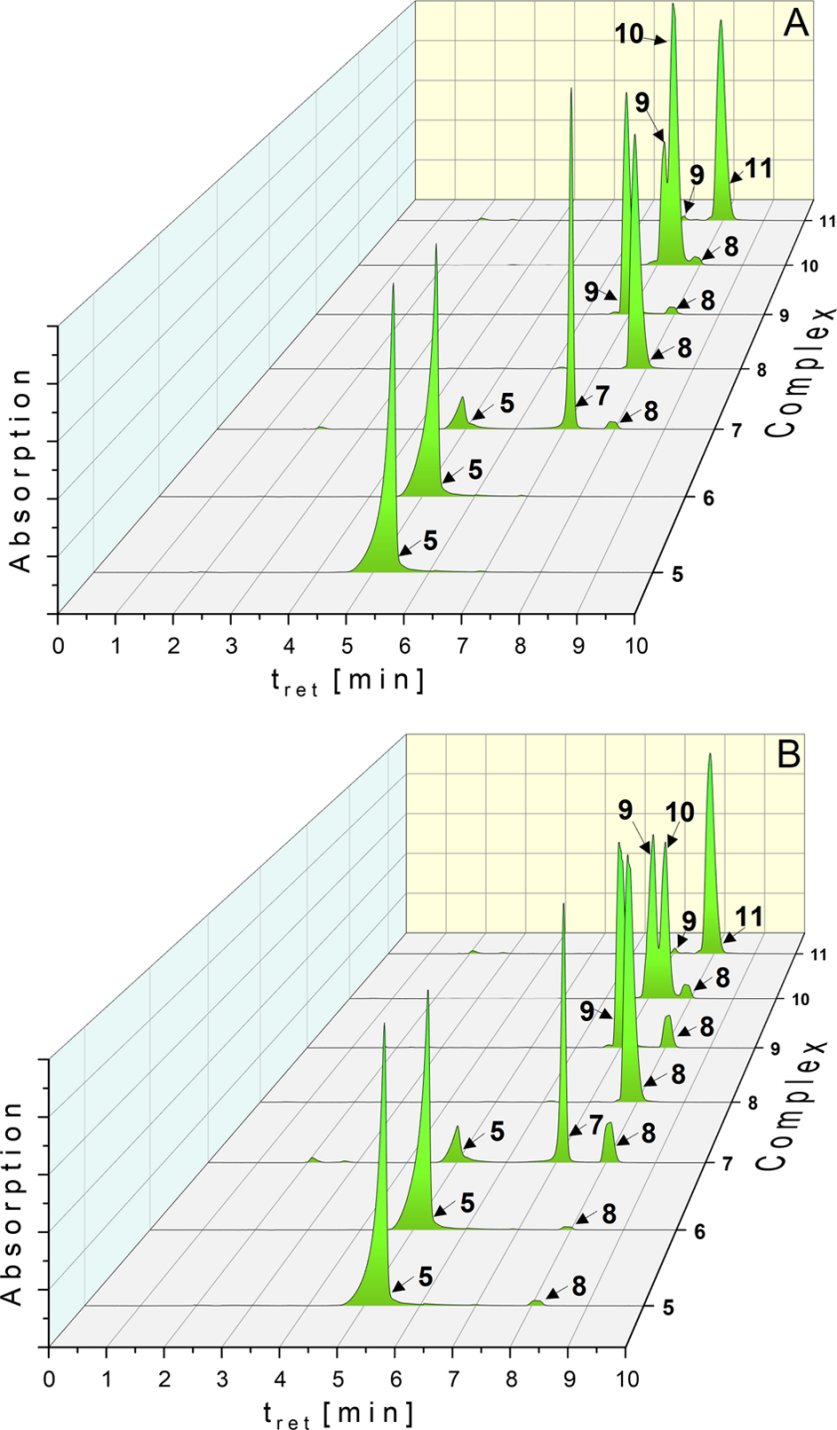
HPLC chromatograms of **5**–**11** (solution: ACN/water = 50/50 (v/v)) in the presence of 6.0 g/L NaCl and 0.4 g/L KCl at *t*_0h_ (A) and *t*_24h_ (B). Complex concentration: 0.5 mM.

A further clue that the peak at *t*_R_ = 5.13 min in the chromatograms of **5** results from (NHC)Au(I)-Cl and not from (NHC)Au(I)-ACN can be driven from Figure 2. High chloride concentrations prevent the substitution of the chloride leaving group by weak nucleophiles. As depicted in Figure 2, only a small amount (0.9%) of **5** underwent ligand scrambling to the [(NHC)_2_Au(I)]^+^ complex **8** during 24 h of incubation. No further peak of a possible (NHC)Au(I)-ACN species was observed.

In contrast, the bromido derivative **6** reacted quantitatively to **5** immediately after mixture of complex and chloride solution (Figure 2A), followed by the same degradation profile as observed for **5** (Figure 2B).

A slightly different reaction behavior showed **7**. At *t*_0h_ (exactly after 1.5 min), 13.3% of **7** converted to **5** and 3.6% to **8** (Figure 2A). After 24 h, 18.9% of **8** was detected. The quantity of **5** stayed constant (Figure 2B).

Incubation of **8** under the same conditions did not result in any degradation. This finding proves that it is the most stable complex and that its formation from (NHC)Au(I)-X complexes **5**–**7** is irreversible.

In the next step, the stability of the gold(III) species **9**–**11** in the presence of chloride was investigated (Figure 2).

Ligand exchange at the dichlorido complex **9** was not observed. However, it was slightly reduced to **8** during 24 h of incubation (by 12.5%).

In contrast, the dibromido derivative **10** underwent preferred substitution reaction to **9** (*t*_0h_: 24.0%, *t*_24h_: 54.4%). The amount of **8** remained nearly constant (*t*_0h_: 2.2%, *t*_24h_: 5.1%) during 24 h.

The iodido ligands at the gold(III) center of **11** stabilized the complex. In chloride containing solution, only traces of **9** (0.4%) were detected at *t*_0h_ without further degradation.

Next, it was evaluated on the example of **7**, whether (NHC)Au(I)-X complexes generally interact with amino acids available in the medium.

**Figure 3:**
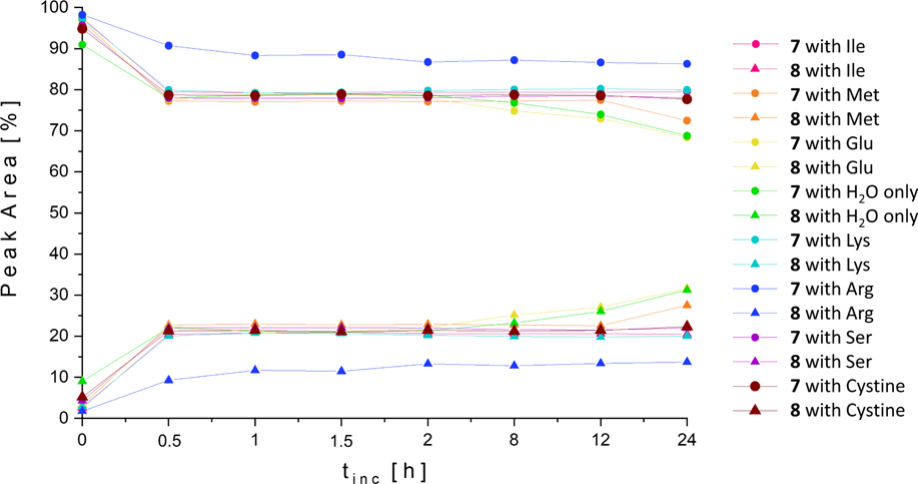
Formation of [(NHC)_2_Au(I)]^+^**8** from **7** in the presence of 20 equiv of non-thiol containing amino acid in ACN/water = 50/50 (v/v). Complex concentration: 0.5 mM.

The HPLC analysis indicated that **7** did not react with the amino acids. Only ligand scrambling to **8** occurred. Time-dependent investigations pointed out that this reaction mostly finished within the first 30 min of incubation (Figure 3). Only in pure ACN/water and in the presence of glutamic acid (Glu) or methionine (Met), a further degradation to **8** took place (*t*_24h_: Glu (31.6%), Met (27.5%), and water (31.2%)).

Interestingly, arginine (Arg) diminished the formation of **8**. After 30 min, the ratio of **7**/**8** was 90/10 and remained stable until 24 h of incubation (Figure 3 and Figure S26).

**Figure 4:**
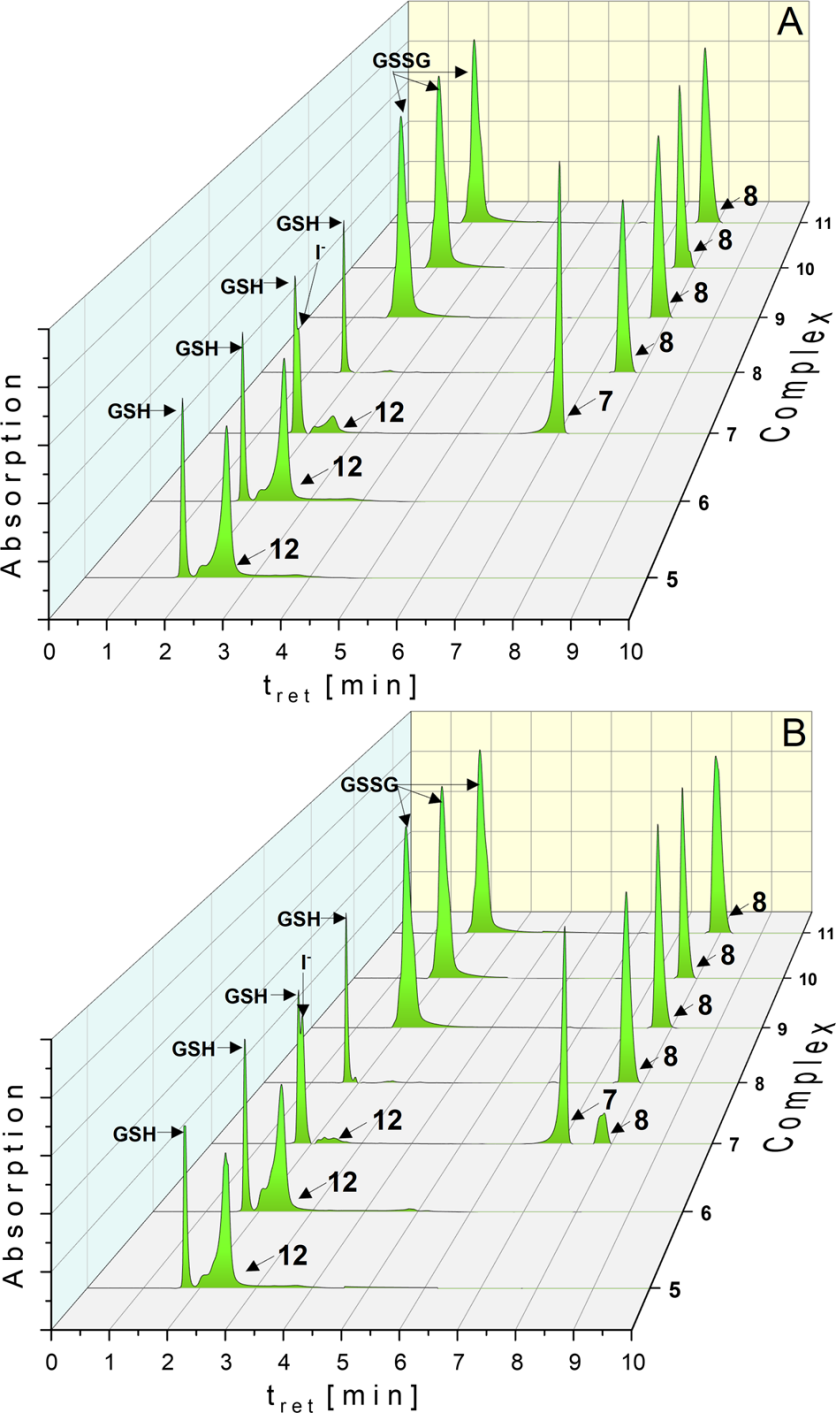
HPLC chromatograms of **5**–**11** in the presence of 20 equiv of GSH (solution: ACN/water = 50/50 (v/v)) at *t*_0h_ (A) and *t*_24h_ (B). Complex concentration: 0.5 mM.

The most important bionucleophile in the medium represents GSH, which is a strong nucleophile and strong reductant due to its cysteine moiety. It was incubated together with the respective complex (**5**–**11**), and the results are depicted in Figure 4.

The used HPLC method allowed the discrimination of GSH from its oxidized form GSSG as well as **5**–**11** from the (NHC)Au(I)-GSH adduct **12**.

The chlorido complex **5**, as well as the bromido complex **6**, reacted quantitatively with GSH in a substitution reaction. Already after 1.5 min (*t*_0h_, Figure 4A), only **12** was observed, without further degradation (*t*_24h_, Figure 4B). Redox reactions did not occur.

In contrast, GSH displaced iodide from **7** (→**12**) only in a proportion of 9.7% (*t*_0h_, Figure 4A), which decreased during 24 h to 4.7%, while 9.8% of **8** was formed (Figure 4B). Time-dependent analysis indicated that ligand scrambling of **7** started after 1 h. At the same time, the amount of **12** diminished (Figure S27).

GSH caused only substitution reactions, without reduction of Au(I) to Au(0). Other redox agents, too, did not affect the (NHC)Au(I)-X complexes. For instance, complex **5** incubated with sodium ascorbate or NADPH for 24 h showed only marginal ligand scrambling to **8** (ascorbate: 3.4%; NADPH: 5.6%, Figures S28 and S29). Neither reduction to gold(0), release of the NHC ligand, nor coordination to the gold(I) center occurred.

In the next step, the influence of reductive agents on the gold(III) complexes **9**–**11** was studied on the example of GSH.

Already the first contact (*t*_0h_) with GSH led to a quantitative reduction to **8** and formation of GSSG (Figure 4).

These results clearly demonstrates that (1) the reduction of [(NHC)_2_Au(III)X_2_]^+^ complexes is independent on the coordinated halides, and (2) the fast reduction to [(NHC)_2_Au(I)]^+^ implicates that **8** might be the biologically active form of **9**–**11**, why examinations in the complete medium are necessary (see below).

The reactions of (NHC)gold(I)-X and [(NHC)_2_Au(III)X_2_]^+^ complexes with GSH were also investigated on the example of **5** and **11** by HR-MS.

Thereto, **5** and GSH were dissolved in MeOH/water mixture (50/50 (v/v)) to a 1/1 proportion and final complex concentration of 100 μM.

**Figure 5:**
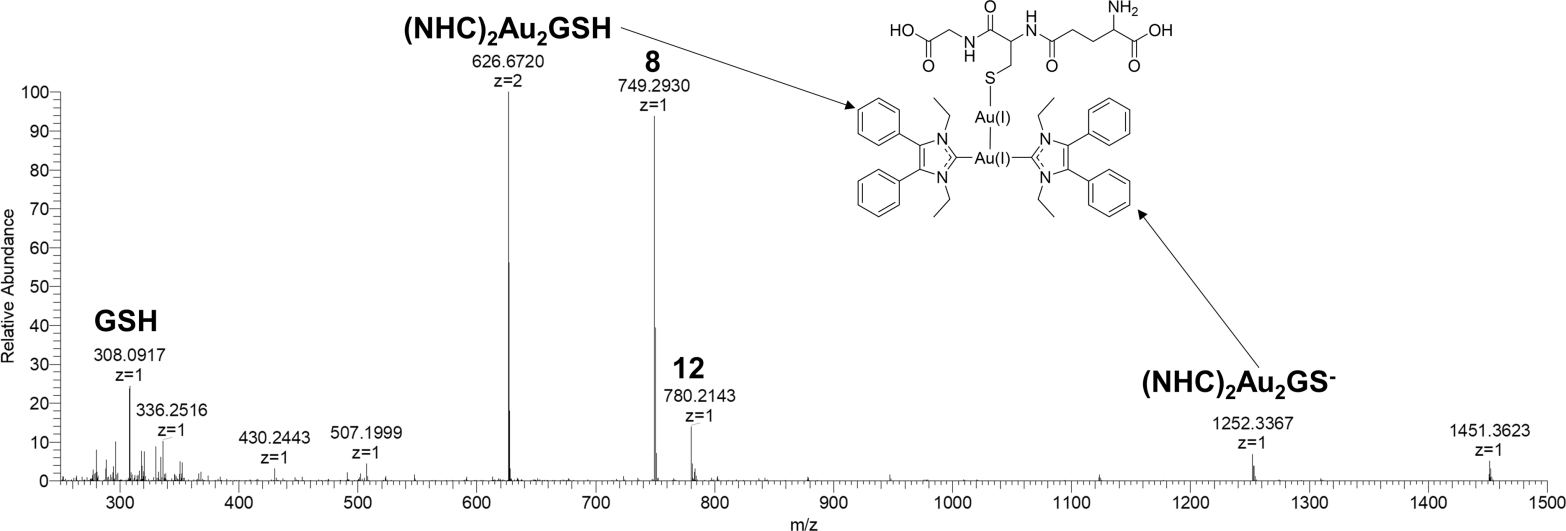
Full mass spectrum obtained from a mixture of **5** in MeOH and GSH in water (50/50, (v/v)) after 24 h.

In addition to the expected reaction product **12** at *m*/*z* 780 (for confirmation via HCD fragmentation see Figure S30), also, higher adducts were detected after 24 h (Figure 5). The doubly charged ion with *m*/*z* 626 ((NHC)_2_Au_2_GSH) and the positive ion with *m*/*z* 1252 ((NHC)_2_Au_2_GS^–^) correspond to a T-shaped intermediate similar to that identified in the water-mediated scrambling reaction of mono-NHC-Au(I)-X complexes.^25^

HCD fragmentation of the ion *m*/*z* 626 (Figure S31) led among others to a species with *m*/*z* 749, which agrees with the [(NHC)_2_Au(I)]^+^ complex **8**, confirming the assumed structure with aurophilic interactions. Table S9 lists all identified ions. These findings document that **12** is capable for ligand scrambling.

Comparable HR-MS studies with **11** and GSH (proportion 1/10, complex conc. 100 μM) were performed. Already after an incubation time of 1.5 min, only signals of **8** (*m*/*z* 749), GSH (*m*/*z* 308), and GSSG (*m*/*z* 613) were observed in the solution, indicating a very fast reduction process (Figure S32).

**Figure 6:**
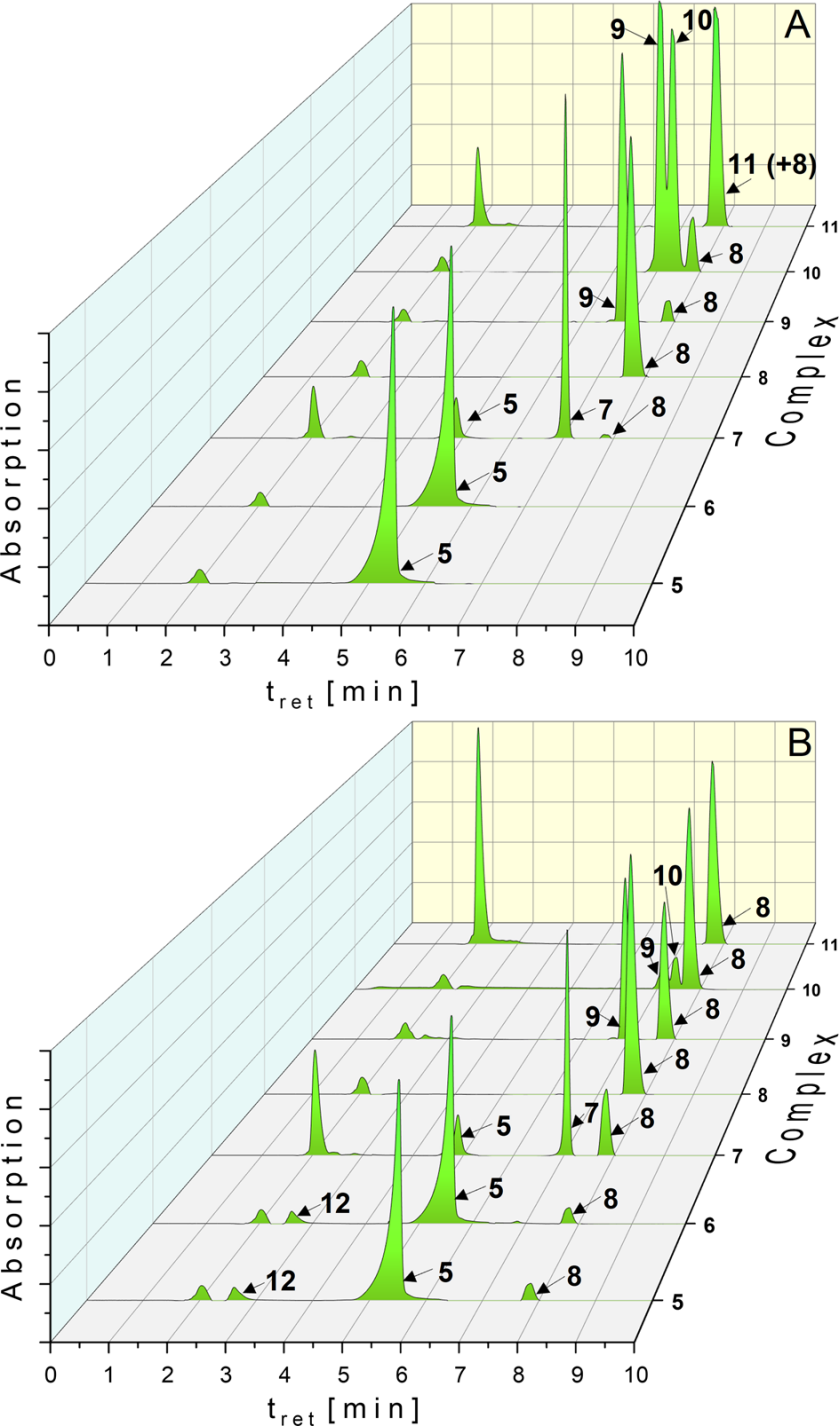
HPLC chromatograms of **5**–**11** (solution: ACN/RPMI 1640 = 50/50 (v/v)) at *t*_0h_ (A) and *t*_24h_ (B). Complex concentration: 0.5 mM.

To study the degradation in the complete cell culture medium, RPMI 1640 (without fetal calf serum (FCS)) was combined with the respective ACN solution of **5**–**11** in a 50/50 (v/v) ratio. Sample preparation and HPLC analysis were performed as described above. The results are depicted in Figure 6.

As expected from the experiment in chloride containing solution, the bromido complex **6** immediately converted to the chlorido complex **5** (*t*_0h_, Figure 6A) and degraded subsequently comparable to **5** (Figure 6B). After 24 h of incubation, **5** only marginally reacted to the GSH-adduct **12** (5.1%) and the [(NHC)_2_Au(I)]^+^ complex **8** (6.2%).

The reduced transformation (compared to the direct reaction with GSH) might be the consequence of the high chloride concentration in the medium. To confirm this assumption, **5** was incubated in a control experiment with 20 equiv of GSH (as described above) in phosphate buffered saline (PBS). At *t*_0h_, 65.7% of **12** was formed. The amount increased during 24 h to 68.0% of **12** and 2.1% of **8** appeared (Figure S33). These results points to an inhibitory effect of chloride on the substitution reaction of (NHC)gold(I)-Cl complexes.

The iodido complex **7** was more stable in the RPMI 1640 medium and transformed at *t*_0h_ only by 14.3% to **5** and by 0.8% to **8**. During 24 h, the amount of **5** remained constant, but **8** increased to 23.1%. No GSH-adduct **12** could be detected.

As expected from the reactivity studies, complex **8** was stable in RPMI 1640 during 24 h of incubation.

The gold(III) complexes **9**–**11** strongly degraded in the medium. Complex **9** was reduced immediately after contact with the medium to **8** by 5.0% and during 24 h by 36.3%.

In case of **10**, additionally, a fast Br/Cl exchange took place. At *t*_0h_, 42.3% of **9** and 9.3% of **8** were formed. During 24 h, the amounts of gold(III) complexes **9** and **10** decreased to 18.2 and 10.7%, respectively, in favor to the gold(I) species **8** (71.1%). In contrast, the solution of **11** contained at *t*_0h_ only the [(NHC)_2_Au(III)I_2_]^+^ complex, which then completely converted to **8** in the redox reaction with GSH within 8 h (Figure 6B and Figure S34).

In a final experiment, the influence of proteins on the degradation and free available (NHC)Au(I)-X complexes was studied on the example of the iodido complex **7**.

The complex (conc. 30 μM) dissolved in RPMI 1640 medium supplemented with 10% FCS was incubated for 4 h at 37 °C in the dark. Subsequently, the proteins were precipitated with ACN and the supernatant was dried by lyophilization. The remaining complexes were extracted from the lyophilizate with DCM. The organic layer was evaporated, and the residue was finally dissolved in 1 mL of ACN. This solution was then analyzed by HPLC as described above.

**Figure 7:**
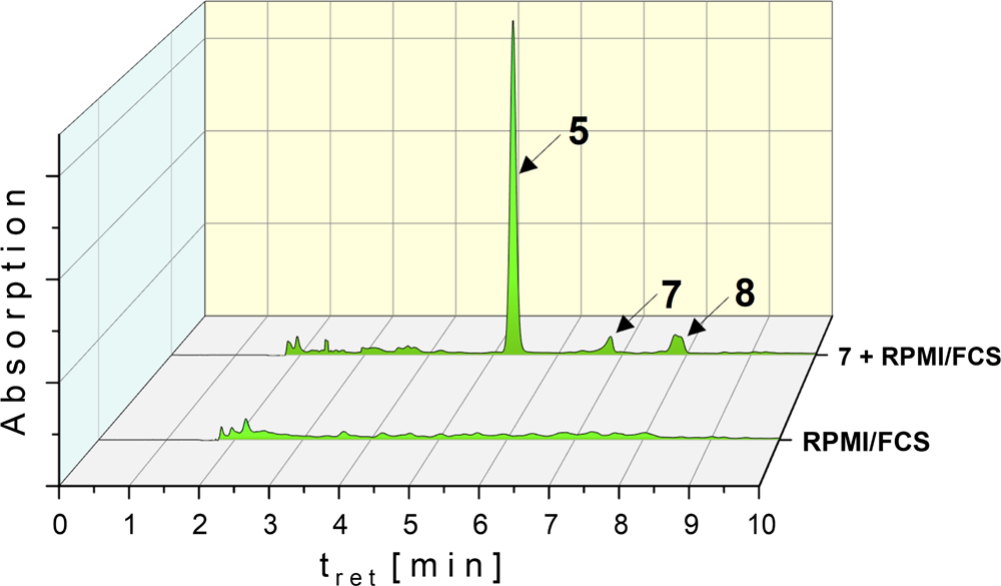
HPLC chromatogram of **7** after 4 h incubation in RPMI 1640 + 10% FCS (complex concentration: 30 μM), protein precipitation, and lyophilization.

It is obvious from Figure 7 that, besides **7**, the chlorido complex **5** and the cationic complex **8** were present as a free fraction. From the peak areas, the concentrations were calculated with 10.12 μM (**5**), 0.78 μM (**7**), and 2.14 μM (**8**). This means that about 50% of the complexes were protein-bound and were separated by protein precipitation with ACN.

In conclusion, the reactivity studies clearly demonstrate that (NHC)Au(I)-X complexes converted in the cell culture medium dependent on the bound halide.

The chlorido complex **5** was very stable in aqueous solution, while the bromido derivative **6** was rapidly and nearly quantitatively transformed to **5**. In both cases, marginal ligand scrambling to the [(NHC)_2_Au(I)]^+^ species **8** took place, whose participation on the biological activity cannot be excluded.

A more complicated degradation profile showed the iodido complex **7**. Besides the initial complex **7**, **5** and **8** were built at concentrations, which make their participation on the biological effects very likely.

The gold(III) complexes **9**–**11** were not stable in the medium. They undergo halide exchange reactions and reduction to **8**.

The [(NHC)_2_Au(I)]^+^ species **8** was the most stable complex among the tested compounds without degradation in the cell culture medium.

### Biological Activity

#### Effects against Wild-Type and Resistant Cancer Cells

*In vitro* cytotoxicity assays were performed to get an insight into the antitumor activity of the (NHC)gold complexes. Complexes **5**–**11**, the established antitumor drug Cisplatin as well as Auranofin, used as a reference for a gold containing drug, were tested in different wild-type and corresponding resistant cancer cell lines. The influence on the metabolic activity was quantified in a modified MTT assay. It involves the conversion of the water-soluble yellow dye MTT (3-(4,5-dimethylthiazol-2-yl)-2,5-diphenyltetrazolium bromide) to an insoluble purple formazan by the action of mitochondrial reductase. Formazan is then solubilized in dimethyl sulfoxide (DMSO), and the concentration is determined by optical density at 570 nm.

The complexes **5** and **6** were low active in the lung cancer cell line A549, the Taxol-resistant subclone A549-R, and chronic myelogenous leukemia(CML) cell line K562 and its Doxorubicin-resistant subclone K562-R (Table 1). The IC_50_ values of both complexes were higher than 10 μM. However, the complexes reduced the viability of human MCF-7 breast cancer cells and the Tamoxifen-resistant subline (MCF-7TamR) with IC_50_ = 4.54–5.76 μM (Table 1). The comparable results of both complexes might be the consequence of the degradation of **6** to **5** in the cell culture medium.

**Table 1:** Metabolic Activity in A549/A549-R, K562/K562-R, and MCF-7/MCF-7TamR Cells Determined in a Modified MTT Assay

	metabolic activity IC_50_^a^ [μM]					
compound	A549	A549-R	K562	K562-R	MCF-7	MCF-7TamR
**5**	>10	>10	>10	>10	5.76 ± 0.88	4.95 ± 0.39
**6**	>10	>10	>10	>10	4.54 ± 0.52	4.64 ± 0.44
**7**	1.12 ± 0.17	1.21 ± 0.36	0.72 ± 0.30	1.53 ± 1.04	0.48 ± 0.08	1.63 ± 0.79
**8**	0.19 ± 0.03	0.22 ± 0.04	0.55 ± 0.26	1.79 ± 0.23	0.16 ± 0.04	0.14 ± 0.02
**9**	0.18 ± 0.01	0.21 ± 0.02	0.55 ± 0.16	1.43 ± 0.29	0.24 ± 0.05	0.26 ± 0.05
**10**	0.20 ± 0.02	0.25 ± 0.04	0.49 ± 0.12	1.59 ± 0.30	0.22 ± 0.07	0.29 ± 0.18
**11**	0.25 ± 0.02	0.27 ± 0.04	0.97 ± 0.26	2.26 ± 1.08	0.19 ± 0.05	0.20 ± 0.07
Auranofin	4.26 ± 0.45	4.42 ± 0.44	3.66 ± 0.27	3.75 ± 0.38	3.15 ± 0.36	1.14 ± 0.46
Cisplatin	6.67 ± 0.63	6.99 ± 0.57	7.91 ± 0.78	9.93 ± 0.77	7.18 ± 1.28	4.13 ± 0.59

a The IC_50_ value represents the concentration causing a 50% decrease in metabolic activity after 72 h of incubation and is calculated as the mean ± SD of two or three independent experiments.

The higher activity in hormone-dependent breast cancer cells points to the carrier function of the 1,3-diethyl-4,5-diphenyl-1*H*-imidazol-2-ylidene moiety as related imidazoles were identified as drugs interfering with the estrogen receptor pathway.^43,44^ This finding will be evaluated more detailed in a forthcoming structure–activity relationship study.

In contrast, the iodido complex **7** showed high potency in A549/A549-R (IC_50_ = 1.12 and 1.21 μM) and K562/K562-R cell lines (IC_50_ = 0.72 and 1.53 μM) and distinctly higher cytotoxicity in MCF-7/MCF-7TamR cells (IC_50_ = 0.48 and 1.63 μM, Table 1), respectively).

The [(NHC)_2_Au(I)]^+^ complex **8** was the derivative, which reduced the metabolic activity most effectively in the low nanomolar range, independent on the cell line used (IC_50_ = 0.14–1.79 μM, Table 1). The complex was 10- to 20-fold higher active than Cisplatin or Auranofin.

Complex **8** is the one with the highest stability, without transformation under cell culture conditions, why the effects can unequivocally be assigned to this species.

The gold(III) derivatives **9**–**11** (IC_50_ = 0.18–2.26 μM, Table 1) were comparably active as **8**. Since it was demonstrated that GSH present in the medium reduces gold(III) to gold(I), it can be assumed that **9**–**11** are prodrugs and **8** was the active ingredient.

**8** was also involved in the activity of **7**. The latter is partially transformed in a ligand scrambling reaction to **8** and in a substitution reaction to **5** as discussed above. Therefore, it is very likely that the effects observed for **7** caused at least three compounds.

The complexes **5**–**11** were further submitted to an extended testing in various ovarian cancer cell lines (Table 2), since related derivatives showed good effects against A2780 ovarian cancer cells, especially Cisplatin-resistant subclones (A2780cis cells).^27,32^

**Table 2:** Metabolic Activity in A2780/A2780cis/A2780V-CSC, IGROV1/IGROV1-CSC Cells Determined in a Modified MTT Assay

	metabolic activity IC_50_^a^ [μM]				
compound	A2780	A2780V-CSC	A2780cis	IGROV1	IGROV1-CSC
**5**	9.45 ± 0.96	9.71 ± 1.20	4.08 ± 0.80	7.53 ± 1.90	9.18 ± 1.32
**6**	9.38 ± 0.80	8.28 ± 0.74	2.87 ± 0.33	5.78 ± 0.90	8.78 ± 0.60
**7**	1.43 ± 0.40	1.65 ± 1.23	0.40 ± 0.06	1.94 ± 0.35	0.47 ± 0.13
**8**	0.18 ± 0.04	0.45 ± 0.07	0.02 ± 0.01	1.04 ± 0.71	0.97 ± 0.66
**9**	0.25 ± 0.04	0.63 ± 0.06	0.05 ± 0.02	0.70 ± 0.38	1.74 ± 0.19
**10**	0.29 ± 0.05	0.76 ± 0.13	0.10 ± 0.04	0.44 ± 0.19	1.29 ± 0.23
**11**	0.28 ± 0.06	1.07 ± 0.21	0.02 ± 0.01	1.28 ± 0.93	2.45 ± 0.97
Cisplatin	6.65 ± 1.42	4.88 ± 0.40	9.80 ± 0.64	2.84 ± 0.76	3.76 ± 0.40

a The IC_50_ value represents the concentration causing a 50% decrease in metabolic activity after 72 h of incubation and is calculated as the mean ± SD of two or three independent experiments.

The cytotoxicity tests included besides the A2780/A2780cis cell lines also those enriched with cells with CSC characteristics (A2780V-CSC and IGROV1-CSC cells).^45^

CSCs are characterized by clonogenicity, asymmetric division, and high tumorigenicity and thus are related to drug resistance and tumor relapse in diverse cancers. Hence, novel inhibitors that eradicate CSCs are promising for mono or combination therapy of refractory and difficult-to-treat cancers.^46^

Auranofin served as a reference and showed a comparable activity in ovarian A2780/A2780cis cancer cells as already published.^19,47^

Compounds **5** and **6** were active with IC_50_ values of 6–9 μM in A2780, A2780V-CSC, IGROV1, and IGROV1-CSC cells and 3–6 μM in A2780cis cells.

Complex **7** showed significantly higher effects due to the transformation in the medium, with the highest cytotoxicity in A2780cis (IC_50_ = 0.40 μM) and IGROV1-CSC cells (IC_50_ = 0.47 μM).

The [(NHC)_2_Au(I)]^+^ complex **8** reduced the metabolic activity of A2780/A2780cis/A2780V-CSC cells with IC_50_ values in the range of 0.02–0.45 μM. It was slightly less active against IGROV1 (IC_50_ = 1.04 μM) and IGROV1-CSC cells (IC_50_ = 0.97 μM). Complexes **9**–**11** showed comparable effects as **8**, indicating again that they act as prodrugs.

The data listed in Table 2 show that A2780cis cells were high-sensitive to **8** and **9**–**11**, with IC_50_ = 0.02–0.10 μM. Cisplatin was 100- to 300-fold less active (IC_50_ = 9.80 μM). Therefore, it seems to be possible to circumvent Cisplatin-resistance with these compounds.

Against A2780 (IC_50_ = 0.18–0.29 μM) and A2780V-CSC (IC_50_ = 0.45–1.07 μM) cells, too, **8**–**11** were more active than Cisplatin (IC_50_ = 6.65 and 4.88 μM), which documents an obvious selectivity for the human ovarian adenocarcinoma.

Especially, the activity in the low nanomolar range against the Cisplatin-resistant subclones is of interest. The resistance of A2780cis cells is triggered by an elevated ability to repair Cisplatin-damaged DNA.^48^

In contrast to Cisplatin, which forms intrastrand cross-links at the DNA, the (NHC)Au(I)-X complexes **5**–**7** can only be mono-functionally bound to nucleobases. This could bypass the DNA repair system.

More difficult is the interpretation of the high cytotoxicity of **8** and its prodrugs **9**–**11**. Complex **8** is inert against substitution reactions, and binding to bionucleophiles seems to be difficult. Nevertheless, Casini et al.^49^ identified the G-quadruplex DNA as binding partner of [(NHC)_2_Au(I)]^+^ complexes. This might be true as mode of action of **8**–**11**.

Further targets are recently discussed by Augello et al.^11^ for the 4-OCH_3_ derivatives of **6** and **8**. Interestingly, although higher antiproliferative, the [(NHC)_2_Au(I)]^+^ complex did not inhibit the thioredoxin reductase, the well-accepted target of (NHC)gold(I) complexes.^16^ Interference into other cellular pathways resulting in an increase of the intracellular ROS (reactive oxygen species) level, which in turn cause DNA damage, could also play an essential role.

These results clearly demonstrate that the complexes **7**–**11** are suitable for the treatment of cells with acquired and intrinsic resistance to common antitumor drugs. Especially, the high effects against CSC-enriched cell lines are noteworthy. Exceptionally, **7** was the most active complex against IGROV1-CSC cells.

Finally, it was evaluated in a preliminary experiment, whether the (NHC)Au(I)-GSH complex **12** possesses cytotoxic effects. It was incubated with A2780 cells and reduced the cell growth with IC_50_ = 15.9 μM, which is about 2-times higher than that of **5** and **6**. Therefore, it is very likely that the GSH adduct is not part of the detoxification system of (NHC)Au(I)-X complexes. Detailed information will be published in a forthcoming paper.

#### Influence on Noncancerous SV-80 Cells

**Figure 8:**
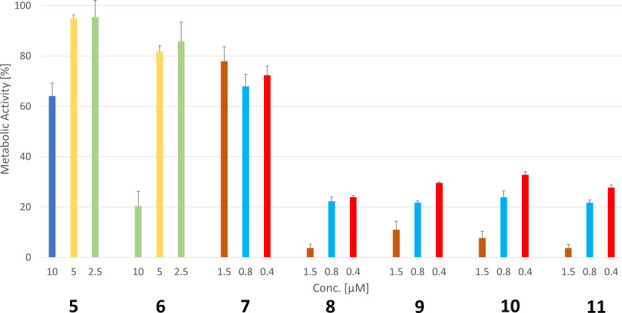
Metabolic activity in noncancerous SV-80 lung fibroblast cells determined in a modified MTT assay and calculated as the mean of three independent experiments.

Finally, **5**–**11** were tested against the noncancerous lung fibroblast cell line SV-80 (Figure 8).

Compounds **5** and **6**, reduced the metabolic activity only at a concentration of 10 μM to 64 and 20%, respectively, while the same effect was achieved with **7** at 5- to 10-fold lower concentrations (metabolic activity 1.5 μM: 77%). Much higher was the influence of the cationic complexes **8**–**11**. A significant reduction of the metabolic activity was already detected at the lowest concentration used (metabolic activity at 0.4 μM: 20–30%).

## Conclusions

Halido[1,3-diethyl-4,5-diphenyl-1*H*-imidazol-2-ylidene]gold(I) and [bis(1,3-diethyl-4,5-diphenyl-1*H*-imidazol-2-ylidene)]dihalidogold(III) hexafluorophosphate (halido = chlorido, bromido, iodido) complexes were synthesized and characterized. Of particular interest was the reactivity of the complexes against components of the cell culture medium and the identification of reaction products. Additionally, the complexes were tested in wildtype and drug-resistant cancer cell lines for antimetabolic activity.

X-Ray structures of the (NHC)Au(I)-X complexes **5**–**7** showed the existence of dimeric units with Au(I)-Au(I) distances of 3.5 to 4.0 Å. Such aurophilic interactions can also be assumed in solution as a prerequisite for the observed ligand scrambling to the [(NHC)_2_Au(I)]^+^ species **8**. Nucleophiles, such as chloride or GSH caused substitution reactions at (NHC)Au(I)-X, in case of the bromido complex **6** even quantitatively to **5** and **12**.

An interaction with non-thiol containing amino acids was not evident. The role of GSH on the cytotoxicity of (NHC)Au(I)-X complexes is not fully elucidated. (NHC)Au(I)-GSH is partially formed in the medium. Interestingly, this reaction did not lead to an inactive species. (NHC)Au(I)-GSH caused antiproliferative effects in A2780 cells, only 2-fold less than **5**.

Regarding the antiproliferative effects of **5**–**11**, it must be mentioned that only **5** and **8** were stable against nucleophiles of the cell culture medium and the biological activity can be ascribed to these species.

The nearly identical activity of **5** and **6** in all cell lines (IC_50_ > 5 μM) resulted from the fast transformation of **6** to **5** in the cell culture medium, while partial ligand scrambling of **7** (→**8**) strongly increased the effects. The formed complex **8** was the most active compound in this study.

In the complete RPMI 1640 medium, supplemented with FCS, protein binding must be taken into account. Quantification of **7** and its degradation products in RPMI 1640/FCS indicated that about 50% of the available complex **7** is located in the protein bound fraction.

The complexes **9**–**11** showed another reaction profile, without ligand scrambling, but with halide exchange reactions.

Most importantly, GSH reduced the gold(III) complexes to **8**, indicating the prodrug character of the [(NHC)_2_Au(III)X_2_]^+^ complexes **9**–**11**.

Complexes **8** and **9**–**11** possessed high cytotoxicity and reduced the viability of, e.g., A2780cis cells in the low nanomolar range. The circumvention of Cisplatin-resistance in ovarian carcinoma cells is therefore feasible with these complexes. Remarkably, the complexes also showed a promising potential to eradicate therapy-resistant CSCs.

Unfortunately, selectivity for tumor cells is not given for **5**–**11**. The growth of SV80 fibroblasts was reduced to the same extent as tumor cells. Therefore, we will use in the following structure–activity relationship study the 1,3-dialkyl-4,5-diaryl-4,5-dihydro-1*H*-imidazol-2-ylidene as a new NHC carrier ligand to design effective (NHC)gold(I) complexes. The results will be presented in forthcoming papers.

## Experimental Section

Chemical reagents and solvents were purchased from commercial suppliers (Sigma-Aldrich, BLDpharm, Fluka, Alfa Aesar, and Abcr) and were used without further purification. Analytical thin-layer chromatography was performed using Polygram SIL G/UV254 (Macherey-Nagel) plastic-backed plates (0.25 mm layer thickness) with fluorescent indicator and Merck TLC Silica gel 60 F254 aluminum-backed plates. The spots were visualized by UV light (254 nm). Column chromatography was done using silica gel 60 (0.040–0.063 mm). NMR spectra were from a Bruker Avance 4 Neo spectrometer (^1^H: 400 MHz, ^13^C: 101 MHz). The center of the solvent signal and the TMS signal were used as internal standards. Deuterated solvents purchased at Eurisotop were used as solvents. HPLC experiments were performed using a Shimadzu prominence HPLC with an autosampler SIL-20A HT, column oven CTO-10AS VP, degassers DGU-20A, detector SPD-M20A, and pumps LC-20 AD with a KNAUER Eurospher 100-5 C18, 250 × 4 mm column. The software used for data processing was LabSolutions. Mass spectra were from an Orbitrap Elite mass spectrometer (Thermo Fisher Scientific, Waltham, MA, USA) using direct infusion and electrospray ionization (ESI). MS data analysis was carried out with Xcalibur. The purity of all tested compounds was >95% as determined by HPLC analysis.

### Synthesis and Characterization

#### Synthesis of the NHC-Ligand

##### 4,5-Diphenyl-1*H*-imidazole (**2**)

Benzil (**1**) (2000 mg) was dissolved in 30 mL of conc. acetic acid together with 15 equiv of ammonium acetate (11.000 mg) and 1.1 equiv of paraformaldehyde (326 mg). The reaction mixture was then refluxed at 118 °C. After 5 h, the solution was neutralized dropwise with saturated aqueous sodium carbonate and extracted three times with ethyl acetate. Removal of the solvent and precipitation with toluene afforded compound **2** (1953 mg, yield 93%) as a yellow solid. ^1^H NMR (400 MHz, DMSO-*d*_6_) δ 12.48 (s, 1H), 7.78 (s, 1H), 7.20–7.49 (m, 10H). ^13^C NMR (101 MHz, DMSO-*d*_6_) δ 135.62, 128.67, 128.16, 127.87, 127.06, 126.35.

##### 1,3-Diethyl-4,5-diphenyl-1*H*-imidazolium Iodide (**3**)

To a stirred solution of **2** (1000 mg) and 1.1 equiv of sodium hydride (120 mg) in 30 mL of anhydrous ACN, 10 equiv of iodoethane (7081 mg, 3.65 mL) was applied and the reaction was stirred under an argon atmosphere at 82 °C for 24 h in the dark. The solvent was removed under reduced pressure, and the residue was taken up in DCM and water. The aqueous phase was separated and washed three times with 20 mL of DCM, and the collected organic phases were dried over sodium sulfate and evaporated *in vacuo* to yield compound **3** (1802 mg, yield 98%) as a brownish solid. ^1^H NMR (400 MHz, DMSO-*d*_6_) δ 9.50 (s, 1H), 7.53–7.39 (m, 10H), 4.11 (q, *J* = 7.3 Hz, 4H), 1.32 (t, *J* = 7.3 Hz, 6H). ^13^C NMR (101 MHz, DMSO-*d*_6_) δ 135.70, 131.90, 131.42, 130.81, 129.69, 125.99, 43.46, 15.42.

##### 1,3-Diethyl-4,5-diphenyl-1*H*-imidazolium Hexafluorophosphate (**4**)

To exchange the iodide counterion for hexafluorophosphate, 1000 mg of **3** was dissolved in 5 mL of MeOH and 5 equiv of potassium hexafluorophosphate (2276 mg) in 5 mL of water was added dropwise. The desired product (**4**) precipitated, and the reaction mixture was stirred at rt for another 24 h. The precipitate was sucked off and washed three times with 10 mL of water. After drying in high vacuum, pure compound **4** (989 mg, yield 95%) was obtained as a yellow solid. ^1^H NMR (400 MHz, DMSO-*d*_6_) δ 9.48 (s, 1H), 7.53–7.39 (m, 10H), 4.11 (q, *J* = 7.3 Hz, 4H), 1.31 (t, *J* = 7.3 Hz, 6H). ^13^C NMR (101 MHz, DMSO-*d*_6_) δ 135.49, 131.71, 131.14, 130.60, 129.49, 125.75, 43.20, 15.13.

#### General Synthesis of Chlorido/bromido(NHC)gold(I) Complexes

The hexafluorophosphate **4** (125 mg) was dissolved under an argon atmosphere and exclusion of light in an anhydrous DCM/MeOH (3 + 3 mL) mixture and supplemented with 0.7 equiv of silver(I)oxide (48 mg). The resulting suspension stirred overnight (12 h) at rt. Then, 5 equiv of lithium chloride/lithium bromide and 1.2 equiv of chlorido(dimethylsulfide)gold(I) (105 mg) were added to the reaction mixture. After stirring for additional 6 h and evaporating of the solvent, the residue was purified by column chromatography on silica gel with DCM/MeOH = 9.8/0.2.

##### Chlorido[1,3-diethyl-4,5-diphenyl-1*H*-imidazol-2-ylidene]gold(I) (**5**)

Complex **5** was synthesized according to the general method with lithium chloride (63 mg) as a chloride source. After chromatography and recrystallization with *n*-pentane, the pure complex **5** (90 mg, yield 60%) was collected as an off-white solid. ^1^H NMR (400 MHz, chloroform-*d*) δ 7.36–7.33 (m, 6H), 7.22–7.19 (m, 4H), 4.21 (q, *J* = 7.2 Hz, 4H), 1.32 (t, *J* = 7.2 Hz, 6H). ^13^C NMR (101 MHz, chloroform-*d*) δ 169.23, 131.10, 130.49, 129.37, 128.87, 127.70, 44.40, 16.92. HR-MS *m*/*z*: calculated for [M – Cl + ACN]^+^ = 514.1557; found 514.1557, calculated for [M – Cl + N_2_]^+^ = 501.1353; found 501.1359. HPLC purity: 99.8%.

##### Bromido[1,3-diethyl-4,5-diphenyl-1*H*-imidazol-2-ylidene]gold(I) (**6**)

Complex **6** was synthesized according to the general method with lithium bromide (129 mg) as a bromide source. Off-white solid, 87 mg (yield 52%). ^1^H NMR (400 MHz, chloroform-*d*) δ 7.38–7.35 (m, 6H), 7.22–7.19 (m, 4H), 4.20 (q, *J* = 7.2 Hz, 4H), 1.31 (t, *J* = 7.2 Hz, 6H). ^13^C NMR (101 MHz, chloroform-*d*) δ 172.83, 131.04, 130.48, 129.36, 128.87, 127.71, 44.30, 16.93. HR-MS *m*/*z*: calculated for [M – Br + ACN]^+^ = 514.1557; found 514.1552. HPLC purity: 99.3%.

##### Iodido[1,3-diethyl-4,5-diphenyl-1*H*-imidazol-2-ylidene]gold(I) (**7**)

**5** (30 mg) dissolved in 5 mL of anhydrous acetone was stirred together with 10 equiv of sodium iodide (88 mg) for 5 min at rt. The solvent was removed under reduced pressure, and the residue was dissolved in DCM and subsequently filtered through a pad of Celite to separate the remaining salts (NaI/NaCl) from **7**. The filtrate was evaporated to dryness, and the residue was recrystallized from *n*-pentane to yield **7** (31 mg, yield 88%) as an off-white solid. ^1^H NMR (400 MHz, chloroform-*d*) δ 7.40–7.33 (m, 6H), 7.22–7.19 (m, 4H), 4.21 (q, *J* = 7.2 Hz, 4H), 1.32 (t, *J* = 7.2 Hz, 6H. ^13^C NMR (101 MHz, chloroform-*d*) δ 179.96, 131.92, 130.48, 129.34, 128.87, 127.73, 44.05, 16.96. HR-MS *m*/*z*: calculated for [M – I + ACN]^+^ = 514.1557; found 514.1548. HPLC purity: 99.8%.

##### Bis[1,3-diethyl-4,5-diphenyl-1*H*-imidazol-2-ylidene]gold(I) Hexafluorophosphate (**8**)

**6** (59 mg) together with 1 equiv of **4** (45 mg) and 2 equiv of potassium carbonate (29 mg) was dissolved in 5 mL of anhydrous acetone under an argon atmosphere. The reaction mixture was stirred for 72 h under protection from light. Subsequently, the solvent was removed under reduced pressure and the crude product was purified by column chromatography (DCM/ethyl acetate = 1/1). Recrystallization from *n*-pentane yielded the pure complex **8** (77 mg, yield 87%) as an off-white solid. ^1^H NMR (400 MHz, chloroform-*d*) δ 7.41–7.33 (m, 12H), 7.28–7.20 (m, 8H), 4.27 (q, *J* = 7.2 Hz, 8H), 1.41 (t, *J* = 7.2 Hz, 12H). ^13^C NMR (101 MHz, chloroform-*d*) δ 182.63, 131.82, 130.60, 129.43, 128.93, 127.47, 44.31, 17.37. HR-MS *m*/*z*: calculated for [M – PF_6_^–^]^+^ = 749.2918; found 749.2919. HPLC purity: 97.9%.

#### Synthesis of (NHC)gold(III) Complexes

##### Bis[1,3-diethyl-4,5-diphenyl-1*H*-imidazol-2-yliden]dichloridogold(III) Hexafluorophosphate (**9**)

**8** (30 mg) was dissolved in 6 mL of DCM, and 10 equiv of dichloroiodobenzene (99 mg) was added. The reaction mixture was stirred at rt for 24 h. The solvent was removed under reduced pressure, and the residue was washed three times with 10 mL of diethyl ether and dried to yield the pure complex **9** (32 mg, yield 98%) as a yellow solid. ^1^H NMR (400 MHz, chloroform-*d*) δ 7.48–7.26 (m, 20H), 4.38 (q, *J* = 7.3 Hz, 8H), 1.48 (t, *J* = 7.2 Hz, 12H). ^13^C NMR (101 MHz, chloroform-*d*) δ 153.26, 133.82, 130.58, 130.19, 129.23, 126.05, 44.04, 16.12. HR-MS *m*/*z*: calculated for [M – PF_6_^–^]^+^ = 819.2296; found 819.2249. HPLC purity: 98.3%.

##### Bis[1,3-diethyl-4,5-diphenyl-1*H*-imidazol-2-ylidene]dibromidogold(III) Hexafluorophosphate (**10**)

To 30 mg of **8**, 1.1 equiv of bromine (6 mg, 2 μL) was added under constant stirring to 6 mL of DCM at −30 °C. Cooling was maintained for 30 min. Subsequently, the reaction mixture was stirred for another 2 h without cooling. The workup was carried out in the same way as described for **9** and yielded pure **10** (33 mg, yield 94%) as an orange solid. ^1^H NMR (400 MHz, chloroform-*d*) δ 7.48–7.27 (m, 20H), 4.35 (q, *J* = 7.3 Hz, 8H), 1.45 (t, *J* = 7.2 Hz, 12H). ^13^C NMR (101 MHz, chloroform-*d*) δ 149.99, 134.19, 130.61, 130.17, 129.21, 126.11, 44.24, 15.74. HR-MS *m*/*z*: calculated for [M – PF_6_^–^]^+^ = 907.1285; found 907.1305. HPLC purity: 99.7%.

##### Bis[1,3-diethyl-4,5-diphenyl-1*H*-imidazol-2-ylidene]diiodidogold(III) Hexafluorophosphate (**11**)

Complex **11** was synthesized according to the procedure described for **10** with the difference of using iodine (10 mg) instead of bromine. Red-orange solid, 37 mg (yield 94%). ^1^H NMR (400 MHz, acetonitrile-*d*_3_) δ 7.47–7.26 (m, 20H), 4.30 (q, *J* = 7.3 Hz, 8H), 1.40 (t, *J* = 7.2 Hz, 12H). ^13^C NMR (101 MHz, acetonitrile-*d*_3_) δ 142.84, 135.50, 131.70, 130.64, 129.51, 127.28, 45.04, 15.12. HR-MS *m*/*z*: calculated for [M – 2I, PF_6_^–^]^+^ = 749.2918; found 749.2872. HPLC purity: 99.1%.

#### HPLC Investigations

Experiments in ACN/water: 1.0 mM solutions of the respective complex were prepared in ACN and diluted with HPLC-grade water (50/50, (v/v)) containing the double-concentrated amount of respective amino acid (20 mM), GSH (20 mM), sodium ascorbate (20 mM), or NADPH (20 mM). As a chloride source, an aqueous solution of KCl 0.8 g/L and NaCl 12 g/L was used. In each case, a final complex concentration of 0.5 mM was achieved. The solutions were incubated for 24 h at rt. For the experiments with RPMI 1640, the aqueous phase was replaced by a cell culture medium.

Samples (20 μL each) were taken at appropriate time points (*t* = 0 (1.5 min), 0.5, 1, 1.5, 2, 8, 12, and 24 h) and analyzed with a Shimadzu prominence HPLC system. The mobile phase consisted of ACN (HPLC-grade) and water (HPLC-grade) with 0.1% TFA. To achieve a separation of the compounds, gradient elution from (70/30 (v/v) to 90/10 (v/v)) of ACN/water (0.1% trifluoroacetic acid (TFA)) was used with a flow rate of 1 mL/min at an oven temperature of 35 °C. All solvents have been degassed before use. The injection volume was 20 μL, and the detection wavelength was set at 235 nm, to detect the gold complexes and the amino acids in the chromatogram. Each experiment was displayed with the program Origin Pro 2018 (Origin LabCorporation, Northampton, MA, USA).

#### ESI-MS Measurements of **5** or **11** with GSH

**5** and **11**, respectively, dissolved in MeOH were combined with an aqueous solution of GSH containing 0.2% formic acid, to get a 50/50 (v/v) solution with a complex concentration of 100 μM and a complex/GSH proportion of 1/1 (**5**) and 1/10 (**11**).

The mixtures were measured on an Orbitrap Elite mass spectrometer (Thermo Fisher Scientific, Waltham, MA, USA) using direct infusion and the HESI source in positive mode after 2 min and 24 h and analyzed with Xcalibur (Thermo Fisher Scientific, Waltham, MA, USA).

#### Determination of the FCS Influence on Stability of **7** in RPMI 1640

To 11 mL of RPMI 1640 including 10% FCS, 11 μL of a freshly prepared 3 mM DMF solution of **7** was added. The solution was then incubated for 4 h at 37 °C in the dark. Proteins were precipitated with 33 mL of ACN. The mixture was cooled to −30 °C and stored for 2 h at −20 °C. Subsequently, the supernatant was separated by centrifugation (3000 rpm), collected, and dried by lyophilization. The lyophilizate was extracted three times with 5 mL of DCM and finally dried under reduced pressure. The residue was taken up in 1 mL of ACN, and 30 μL was analyzed by the HPLC method described above. Peak area was used for quantification. The calibrations of **5**, **7**, and **8** were obtained by injecting 30 μL of the respective solution of pure substances in ACN at a concentration of 1–30 μM (*R*^2^ > 0.99).

#### Crystallography

A Bruker D8 Quest Kappa diffractometer equipped with a Photon 100 detector was used to collect the single-crystal intensity data. Monochromatized MoKα radiation was generated by an Incoatec microfocus X-ray tube (50 kV/1 mA power settings) in combination with a multilayer optic. The supplementary crystallographic data were deposited as CCDC 2180314-2180320 (**5**–**11**). Copies of the data can be obtained, free of charge, at the Cambridge Crystallographic Data Centre.

#### Biological Methods

##### Cell Lines

The ovarian carcinoma cell lines A2780 and A2780cis were kindly provided by the Department of Gynecology, Medical University Innsbruck. To maintain resistance, A2780cis cells were incubated every second week with Cisplatin (1 μM). The resistance of the A2780cis cells is caused by an increased ability to repair DNA damage mediated by cytogenetic abnormalities.^48^ The fibroblast cell line SV-80, the lung cancer cell lines A549 and A549-R, and the ovarian cancer cell lines IGROV1, IGROV1-CSC, and A2780V-CSC were kindly provided by the Department of Internal Medicine V, Medical University Innsbruck. A549-R cells are resistant against Paclitaxel (5 nM). The resistance was generated by cultivating the A549 cells with gradually increasing concentrations of Paclitaxel. IGROV1-CSC and A2780V-CSC (originally termed IGROV1 SP and A2780V SP) represent therapy resistant cell lines with an enriched proportion of cancer cells with stem cell characteristics.^45^ The CML cell line K562-R cells was originally described by Hui et al. as a Doxorubicin-resistant subclone of the K562 cell line (originally termed as KD225).^50^ K562-R cells are also resistant toward Imatinib, as we described in previous publications.^51−54^

The breast cancer cell lines MCF-7 and MCF-7TamR were purchased from DSMZ, German Collection of Microorganisms and Cell Cultures, Braunschweig, Germany. To maintain Tamoxifen resistance, the MCF-7TamR cells were treated with Tamoxifen (1 μM) every two weeks.

The cell lines A2780, A2780cis, A2780V-CSC, SV-80, A549, A549-R, K562, K562-R, IGROV1, and IGROV1-CSC were cultivated in RPMI 1640 without phenol red (BioWhittaker, Lonza, Walkersville, MD, USA), supplemented with l-glutamine (2 mM) and FCS (10%) (all from Invitrogen Corporation, Gibco, Paisley, Scotland) at 37 °C in a 5% CO_2_/95% air atmosphere and fed/passaged twice weekly. The cell lines MCF-7 and MCF-7TamR were cultivated in DMEM without phenol red (PAN Biotech, Aidenbach, Germany) and supplemented with sodium pyruvate (100 mM) (PAN Biotech, Aidenbach, Germany) and FCS (10%) (Invitrogen Corporation, Gibco, Paisley, Scotland) under the same conditions as the other cell lines.

##### Analysis of Cell Growth Inhibition

Exponentially growing cells were seeded at a density of 1500 cells/well (A2780, A2780cis, A2780V-CSC, A549, and A549-R), 1750 cells/well (MCF-7 and MCF-7TamR), 2500 cells/well (IGROV1 and IGROV1-CSC), 3000 cells/well (SV-80), and 20,000 cells/well (K562 and K562-R), respectively, into clear flat-bottom 96-well plates in triplicates. After 24 h of incubation for adherent cell lines and 1 h of incubation for suspension cell lines (K562 and K562-R) at 37 °C in a humidified atmosphere (5% CO_2_/95% air), the compounds were added to reach the indicated concentrations, respectively. The indicated final concentrations of the compounds in the well are 20, 10, 5, 2.5, 1.25, and 0.625 μM for substances **5** and **6** and the references Cisplatin and Auranofin, as well as 1.5, 0.75, 0.375, 0.1875, 0.09375, and 0.046875 μM for substances **7**–**11**. All stock solutions were prepared in DMF at a concentration of 10 mM and were then diluted with RPMI 1640, supplemented with l-glutamine (2 mM) and FCS (10%), to the respective concentrations. After another 72 h of incubation, the cellular metabolic activity was measured employing a modified 3-(4,5-dimethylthiazol-2-yl)-2,5-diphenyltetrazolium bromide (MTT) assay (EZ4U kit, Biomedica, Vienna, Austria) following the manufacturer’s protocol. The optical density of the particular medium was subtracted to exclude the unspecific staining caused by FCS containing medium. The values were calculated with Excel 2019 (Microsoft, Redmond, WA, USA) using nonlinear regression and decal logarithm of the inhibitor versus variable slope equation, while the top constraint was set to 100%.

## Data Availability

Authors will release the atomic coordinates upon article publication.

## Supporting Information Available

^1^H and ^13^C NMR spectra of **5**–**11**; HPLC chromatograms of **5**–**11** recorded in ACN, **7** recorded in ACN/water in the presence of amino acids and GSH, **11** recorded in ACN/RPMI 1640, **5** recorded in ACN/PBS in the presence of GSH, and **5** recorded in ACN/water in the presence of NADPH or sodium ascorbate; HR-MS data of experiments with GSH and **5** or **11**; ORTEP of **6**, **7**, and **10**–**11**; crystallographic data of **5**–**11** (PDP IDs: 2180314 (**5**), 2180315 (**6**), 2180316 (**7**), 2180317 (**8**), 2180318 (**9**), 2180319 (**10**), 2180320 (**11**) (PDF)
Molecular formula strings of **5**–**11** with IC_50_ values in A549, A549-R, K562, K562-R, MCF-7, MCF-7TamR, A2780, A2780cis, A2780V-CSC, IGROV1, and IGROV1-CSC cell lines (CSV)

## Abbreviations

ACN: acetonitrile
CML: chronic myelogenous leukemia
CSC: cancer stem cells
DCM: dichloromethane
DMF: dimethyl formamide
DMSO: dimethyl sulfoxide
FCS: fetal calf serum
ESI: electrospray ionization
equiv: equivalents
HPLC: high-performance liquid chromatography
HR-MS: high-resolution mass spectrometry
MeOH: methanol
MTT: 3-(4,5-dimethylthiazol-2-yl)-2,5-diphenyltetrazolium bromide
NHC: *N*-heterocyclic carbene
NMR: nuclear magnetic resonance
PhICl_2_: dichloroiodobenzene
ppm: parts per million
RP: reversed phase
rpm: rounds per minute
RPMI: Roswell Park Memorial Institute
rt: room temperature
TamR: Tamoxifen resistant
TFA: trifluoroacetic acid
(v/v): volume per volume.
