# Supplementary material for: Reaction Behavior of [1,3-Diethyl-4,5-diphenyl-1H-imidazol-2-ylidene] Containing Gold(I/III) Complexes against Ingredients of the Cell Culture Medium and the Meaning on the Potential Use for Cancer Eradication Therapy
Source: J Med Chem. 2023 Jun 9;66(12):8238–50. doi: 10.1021/acs.jmedchem.3c00589 (PMC10291549; doi:10.1021/acs.jmedchem.3c00589)
Supplement: Supplementary file 1 — jm3c00589_si_001.pdf [file jm3c00589_si_001.pdf]

Supporting information for:

Reaction behavior of [1,3-diethyl-4,5-diphenyl-1*H*-imidazol-2-ylidene] containing gold(I/III) complexes against ingredients of the cell culture medium and the meaning on the potential use for cancer eradication therapy

Paul Kapitza,[†,#] Amelie Scherfler,[†,#] Stefan Salcher,[‡] Sieghart Sopper,[‡] Monika Cziferszky,[†] Klaus Wurst,[§] and Ronald Gust[*,†]

[†]Department of Pharmaceutical Chemistry, Institute of Pharmacy, Center for Molecular Bioscience Innsbruck, University of Innsbruck, Innrain 80/82, A-6020 Innsbruck.

[‡]Department of Internal Medicine V, Hematology and Oncology, Medical University Innsbruck, Anichstrasse 35, A-6020 Innsbruck.

[§]Department of General, Inorganic and Theoretical Chemistry, University of Innsbruck, Innrain 80/82, A-6020 Innsbruck.

Corresponding Author
Ronald Gust; gust.ronald@gmail.com.

Table of content:

1. Characterization of **5-11** S2
   1.1 HPLC chromatograms S2
   1.2 ^{1}H NMR spectra S5
   1.3 ^{13}C NMR spectra S9
2. X-ray structures of **6**, **7**, **10**, and **11** S12
3. Crystallographic data and comparison of gold-related crystal parameters of **5-11** S14
4. Reaction of **7** with non-thiol containing amino acids S22
5. HPLC chromatograms of **7** with 20 eq. GSH S23
6. HPLC chromatograms of **5** with 20 eq. of NADPH or ascorbate S23
7. HR-MS experiments with **5** or **11** and GSH S24
8. HPLC chromatograms of **5** with 20 eq. GSH in PBS S25
9. HPLC chromatograms of the conversation from **11** to **8** in the presence of RPMI 1640 ...... S26

1. Characterization of **5-11**

1.1 HPLC chromatograms

Complexes **5-11** (approximately 0.5 mg) were dissolved in 0.75 ml of ACN (HPLC-grade) and 30 µl were injected to a Shimadzu prominence HPLC with autosampler SIL-20A HT, column oven CTO-10AS VP, degassers DGU-20A, detector SPD-M20A, and pumps LC-20AD. As column a KNAUER Eurospher 100-5 C18, 250 x 4 mm was used. Gradient elution (70/30 (v/v) to 90/10 (v/v)) of ACN/water (0.1% TFA) was used and the software LabSolutions was used for data processing. Purity was calculated using the peak area %.

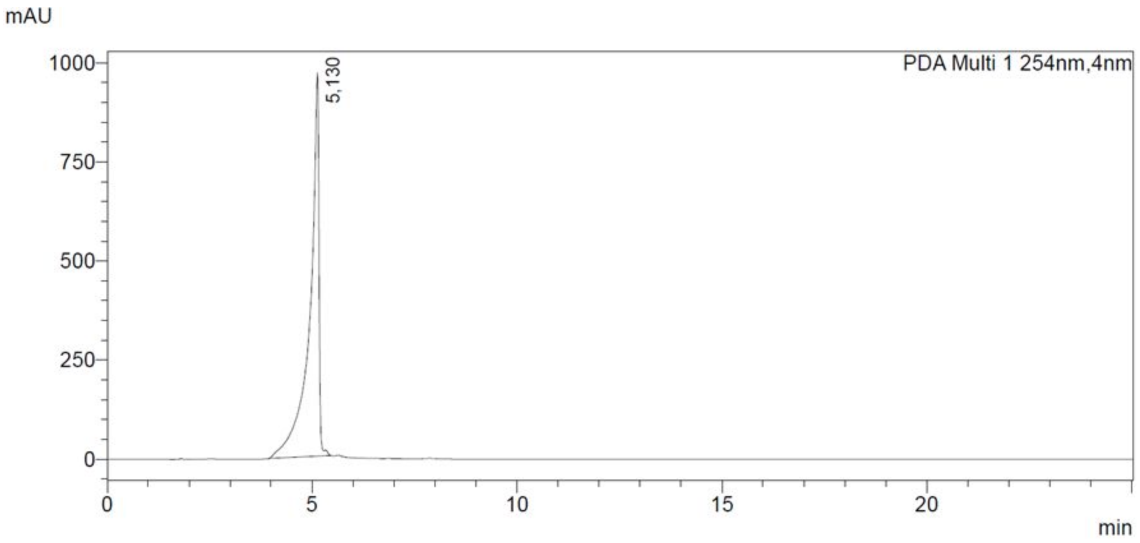

Figure S1. HPLC chromatogram of **5** dissolved in ACN (purity: 99.8%).

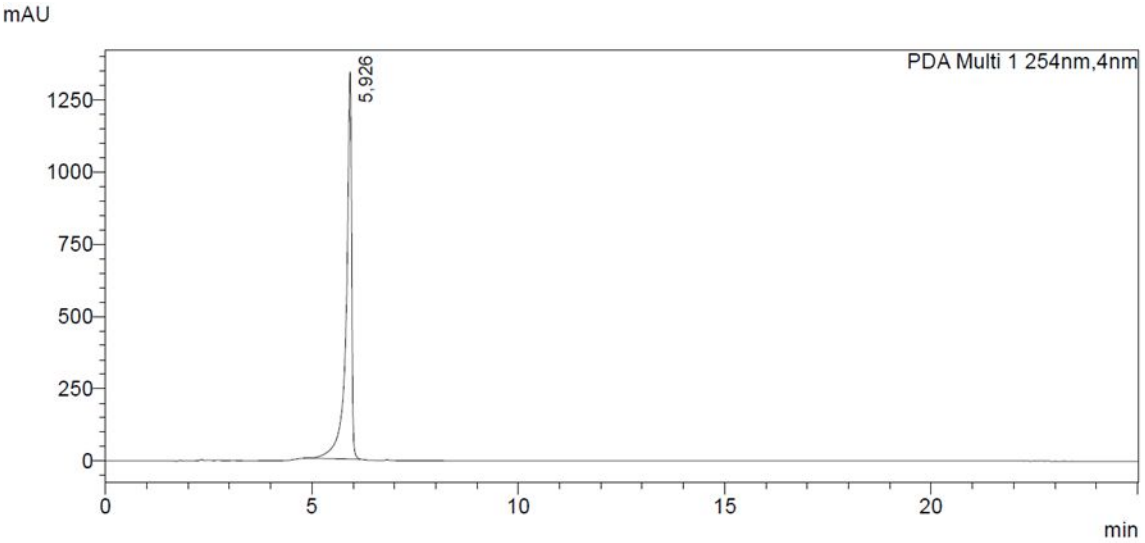

Figure S2. HPLC chromatogram of **6** dissolved in ACN (purity: 99.3%).

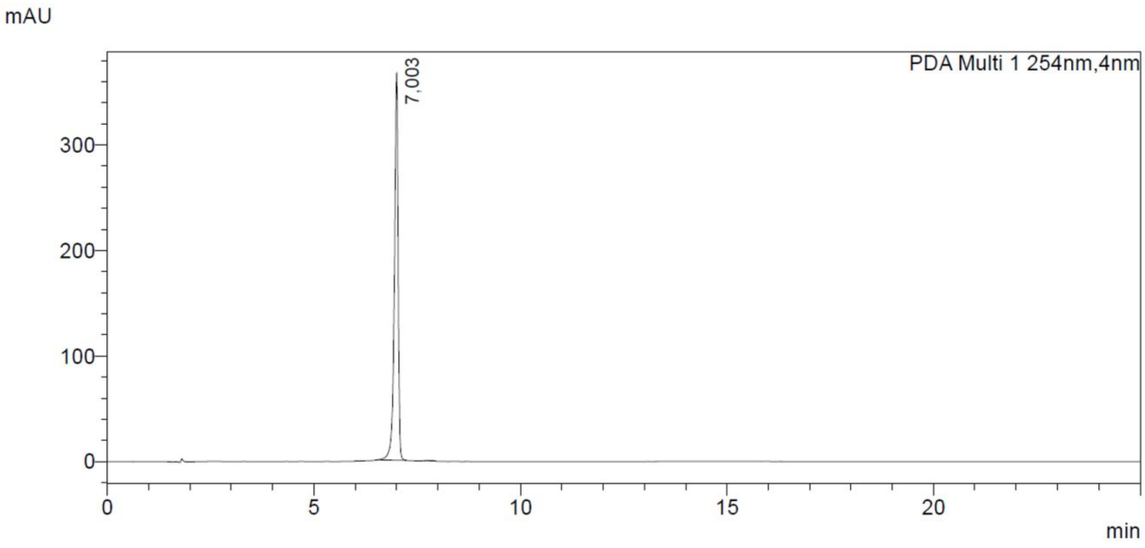

Figure S3. HPLC chromatogram of **7** dissolved in ACN (purity: 99.8%).

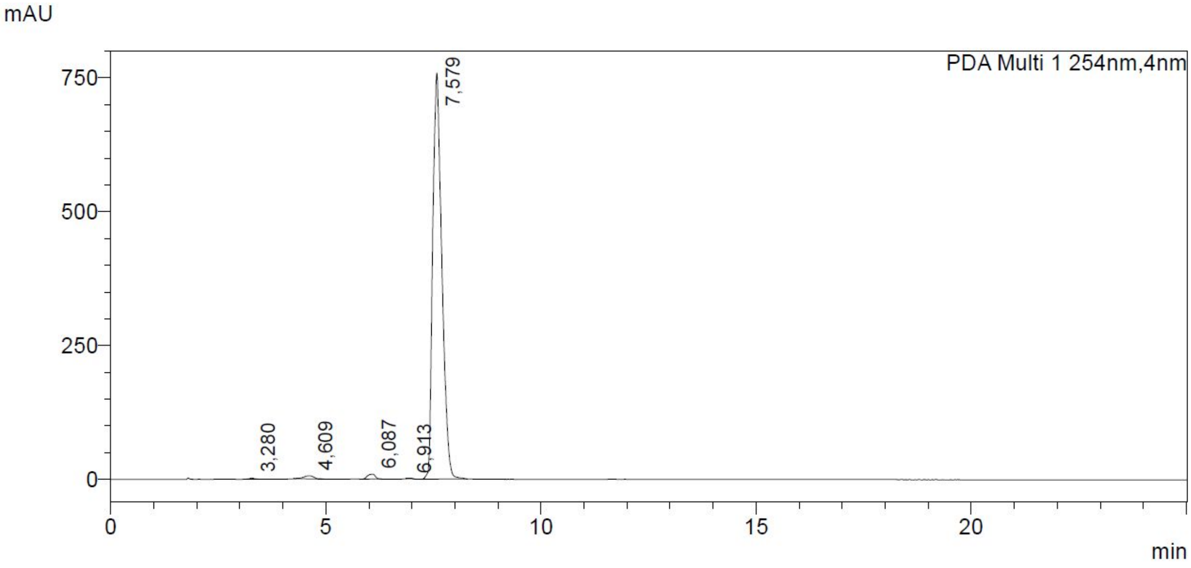

Figure S4. HPLC chromatogram of **8** dissolved in ACN (purity: 97.9%).

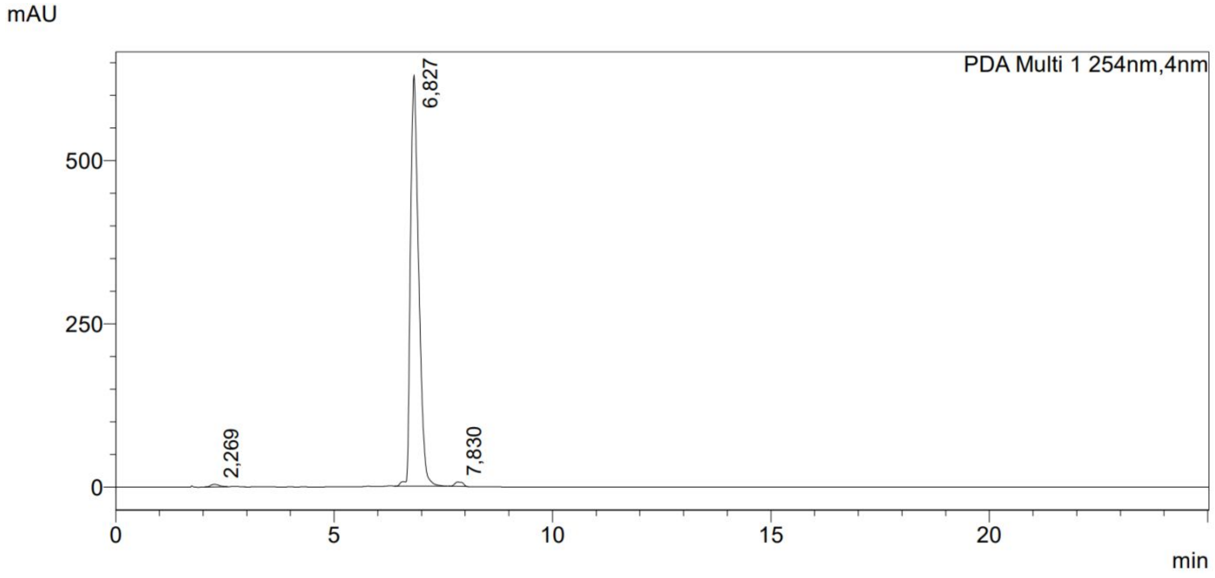

Figure S5. HPLC chromatogram of **9** dissolved in ACN (purity: 98.3%).

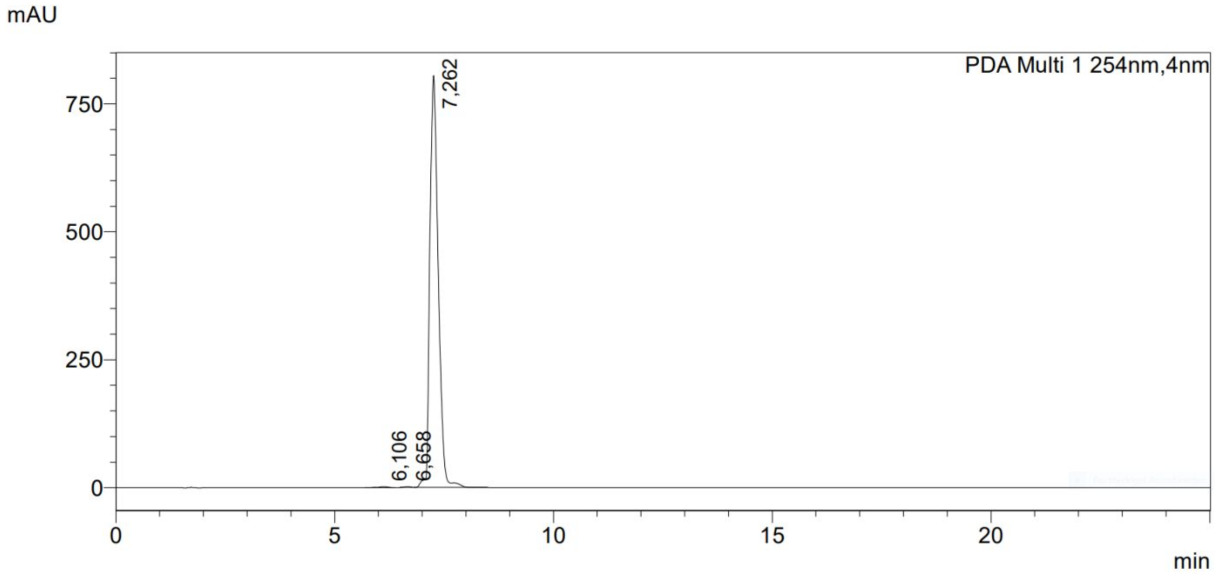

Figure S6. HPLC chromatogram of **10** dissolved in ACN (purity: 99.7%).

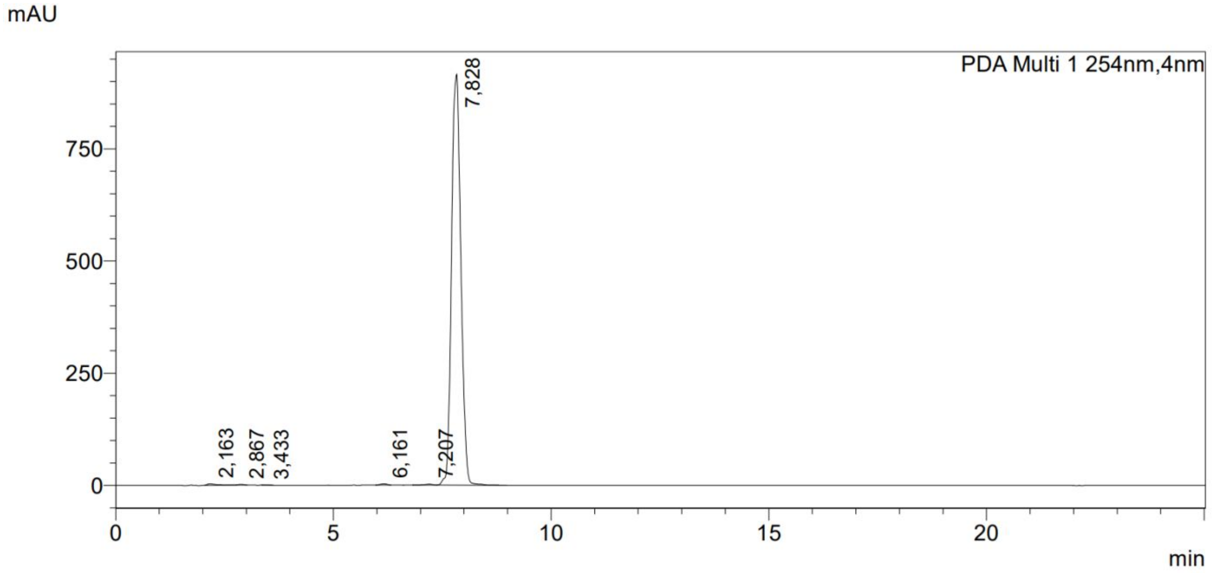

Figure S7. HPLC chromatogram of **11** dissolved in ACN (purity: 99.1%).

1.2 ^{1}H NMR spectra

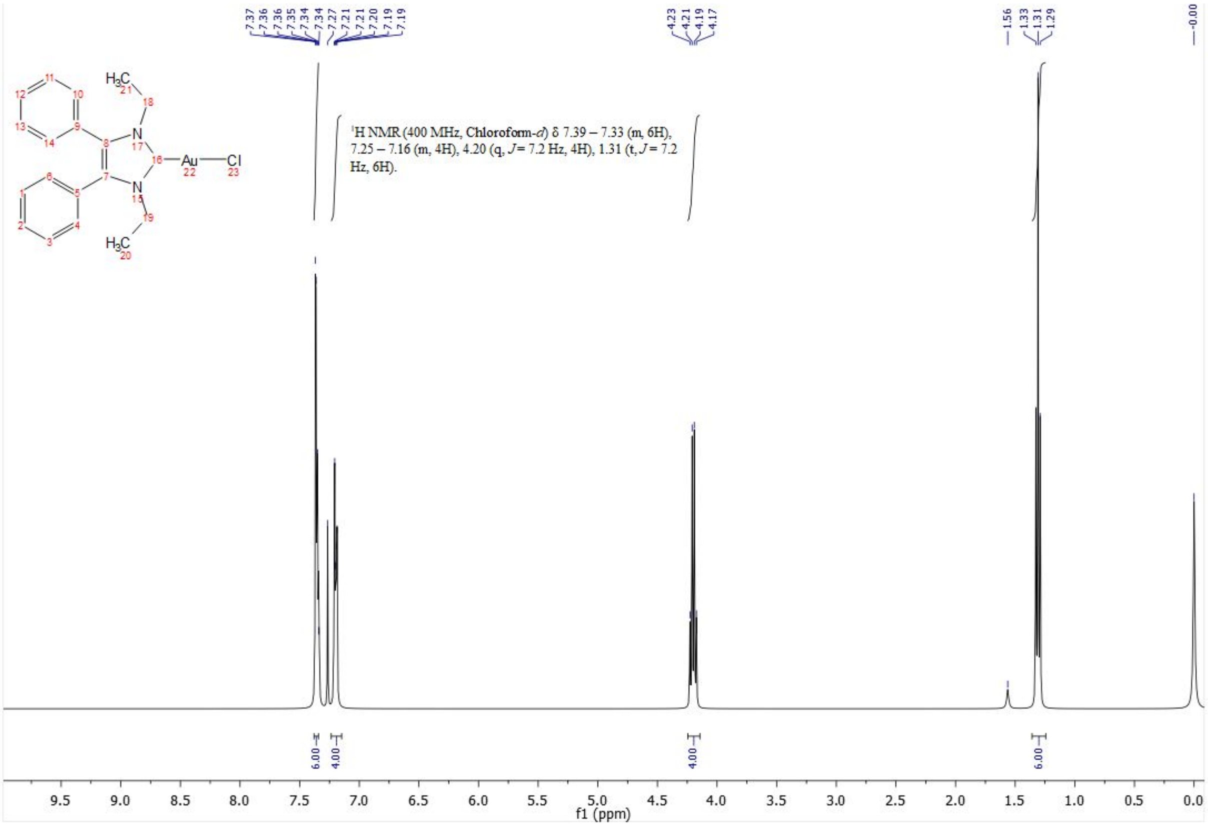

Figure S8. ^{1}H NMR spectrum (400 MHz) of **5** recorded in chloroform-*d*.

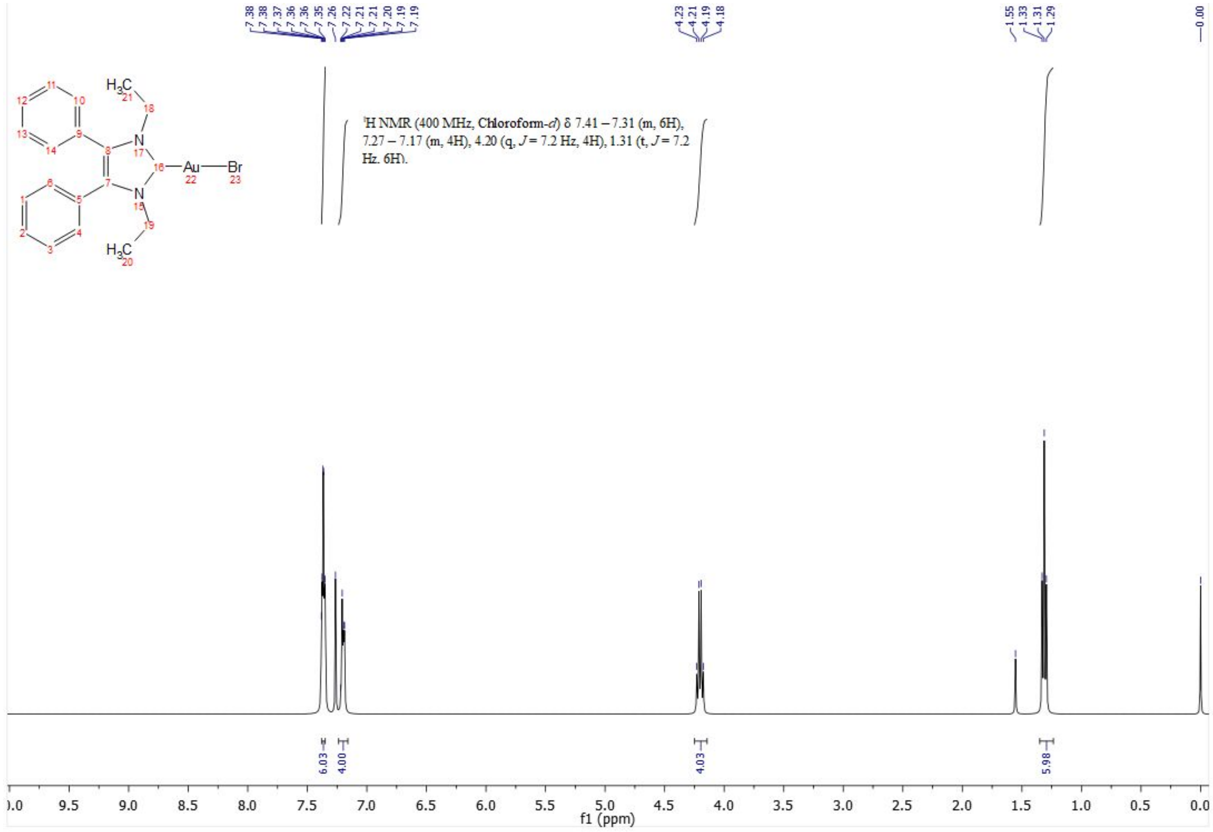

Figure S9. ^{1}H NMR spectrum (400 MHz) of **6** recorded in chloroform-*d*.

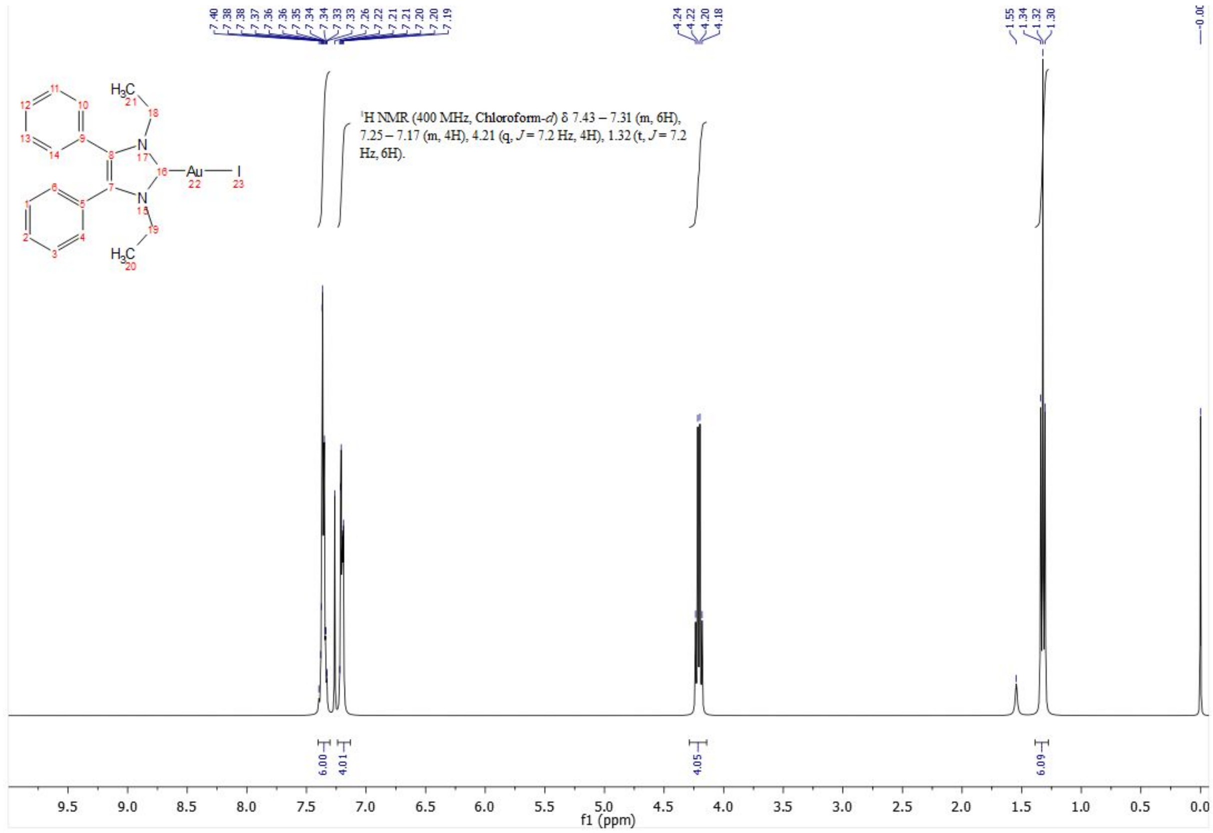

Figure S10. ^{1}H NMR spectrum (400 MHz) of **7** recorded in chloroform-*d*.

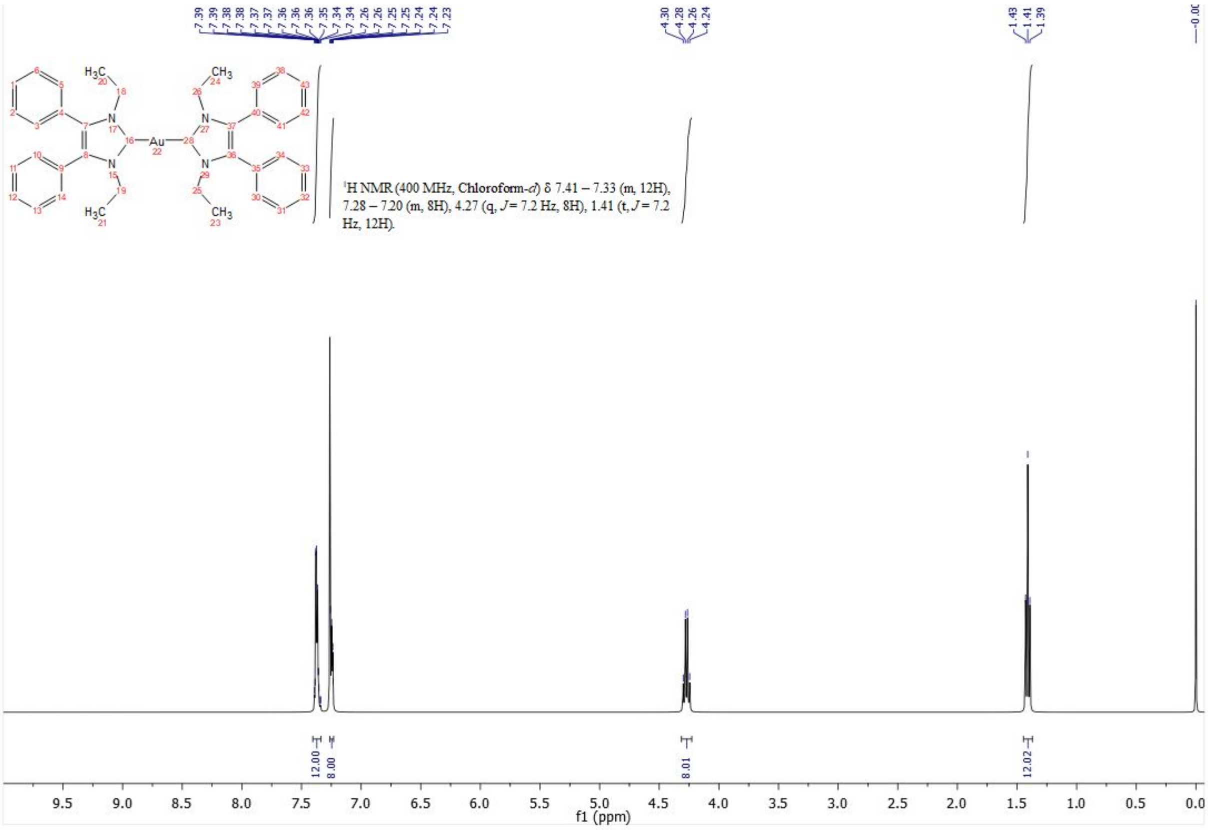

Figure S11. ^{1}H NMR spectrum (400 MHz) of **8** recorded in chloroform-*d*.

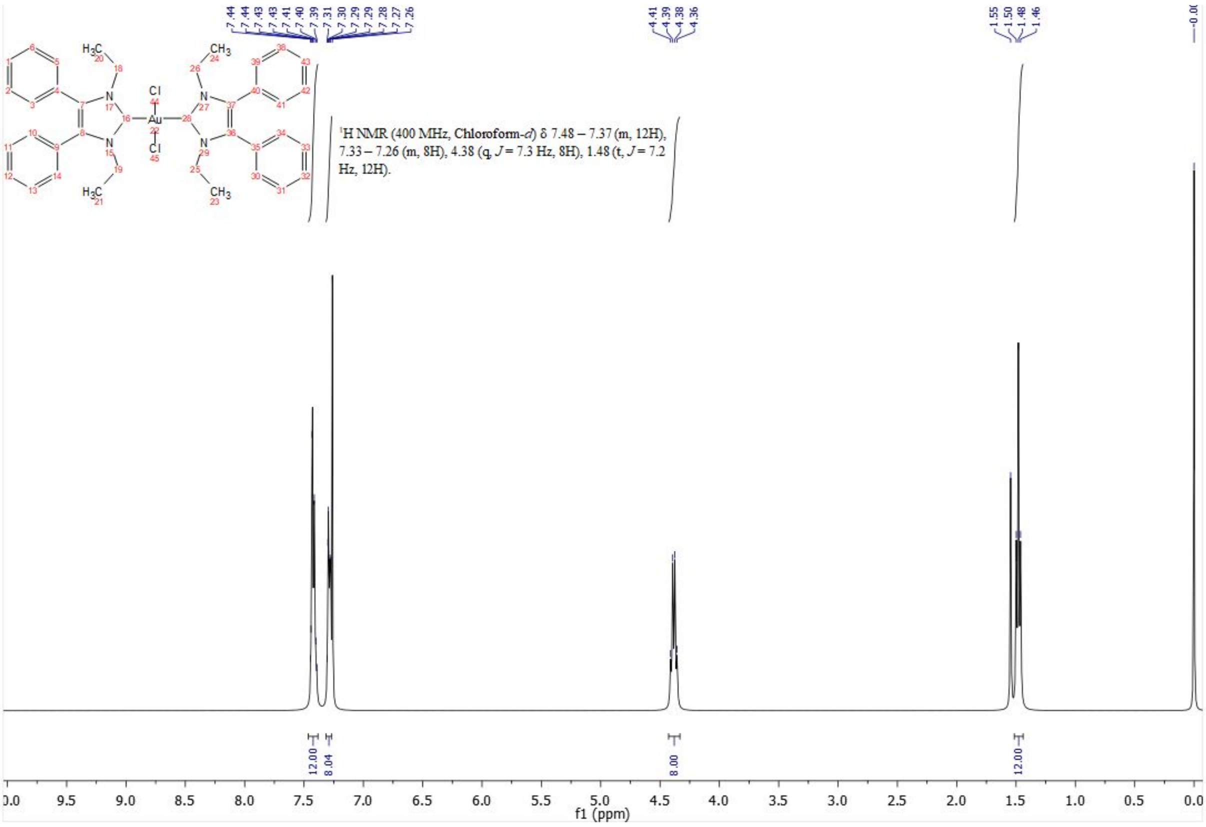

Figure S12. ^{1}H NMR spectrum (400 MHz) of **9** recorded in chloroform-*d*.

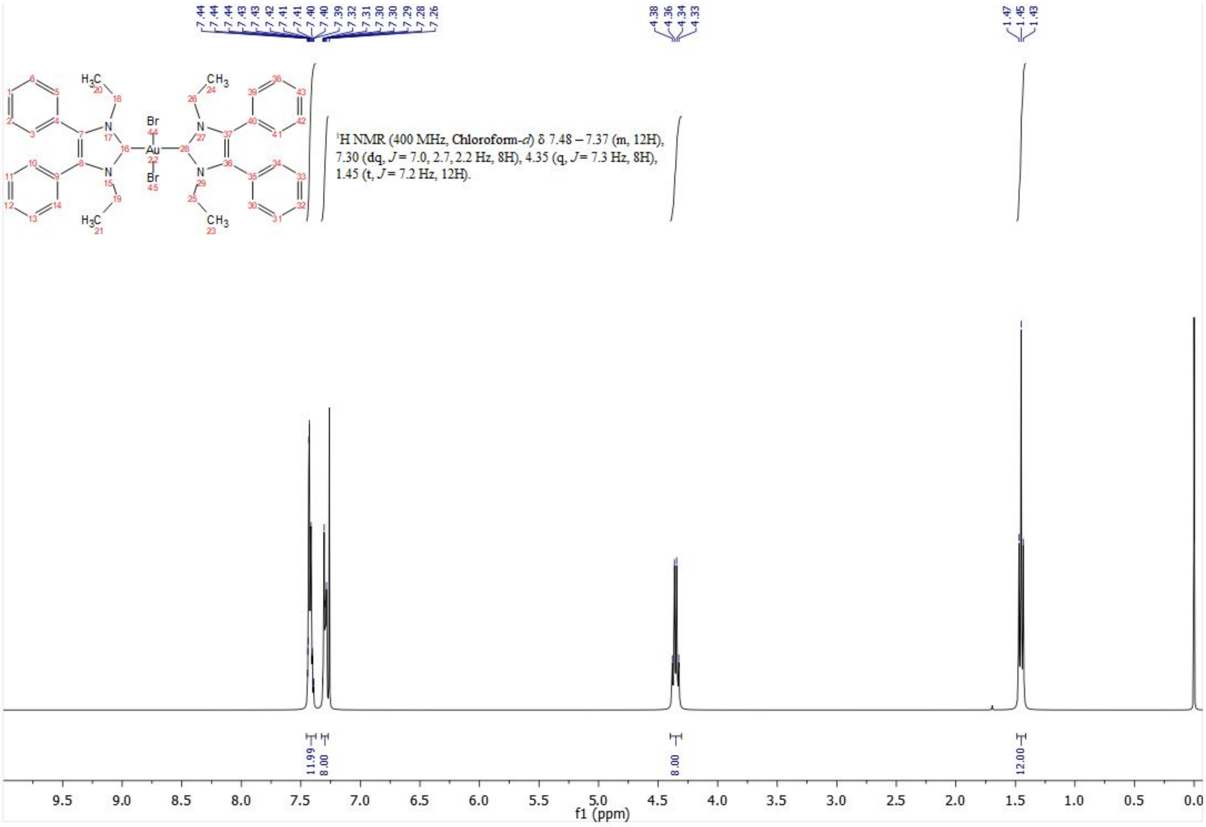

Figure S13. ^{1}H NMR spectrum (400 MHz) of **10** recorded in chloroform-*d*.

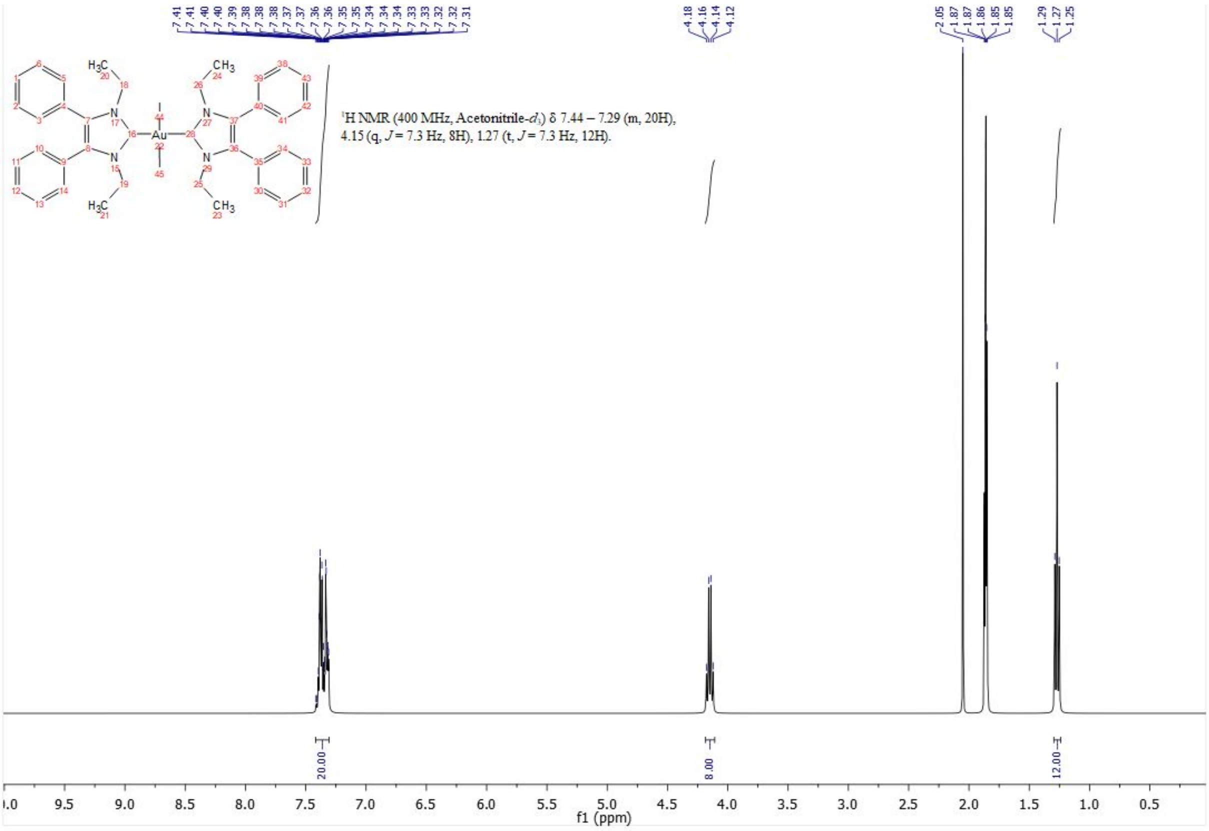

Figure S14. ^{1}H NMR spectrum (400 MHz) of **11** recorded in acetonitrile-*d*$_{3}$.

1.3 ^{13}C NMR spectra

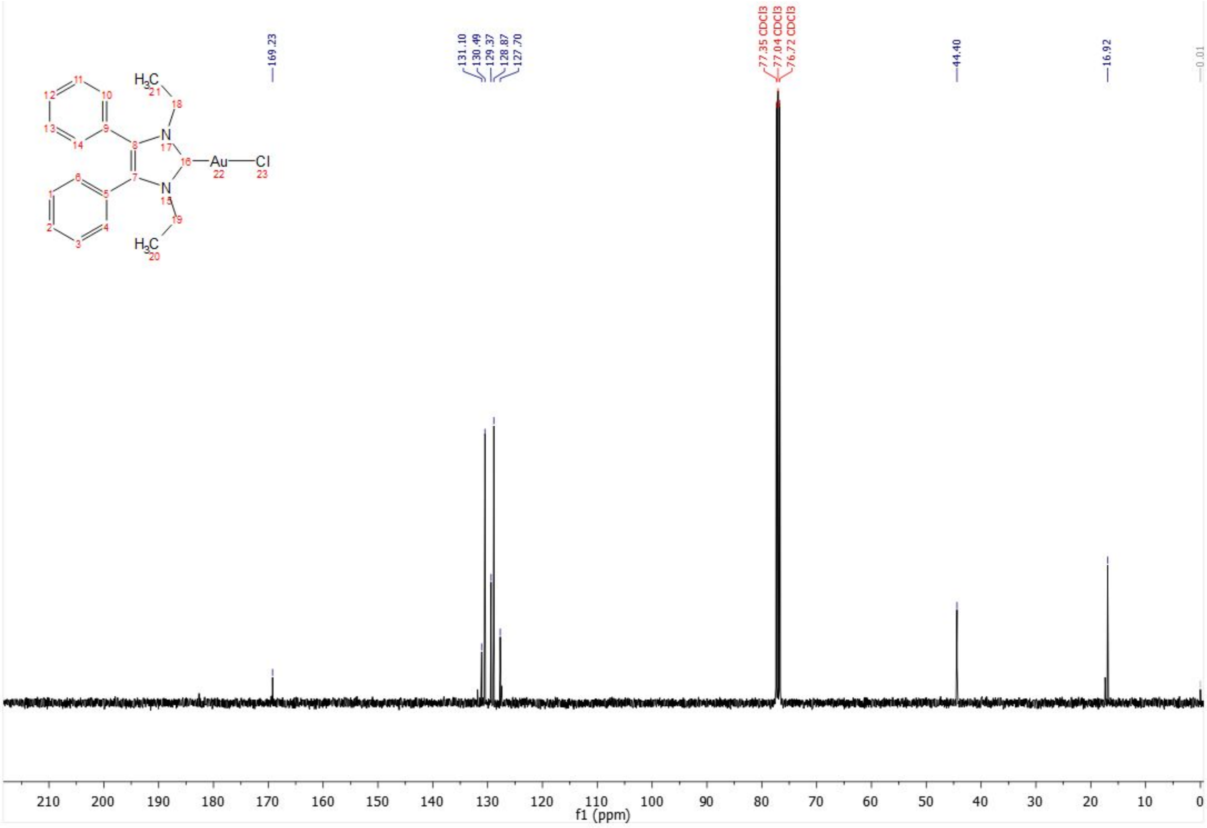

Figure S15. ^{13}C NMR spectrum (101 MHz) of **5** recorded in chloroform-*d*.

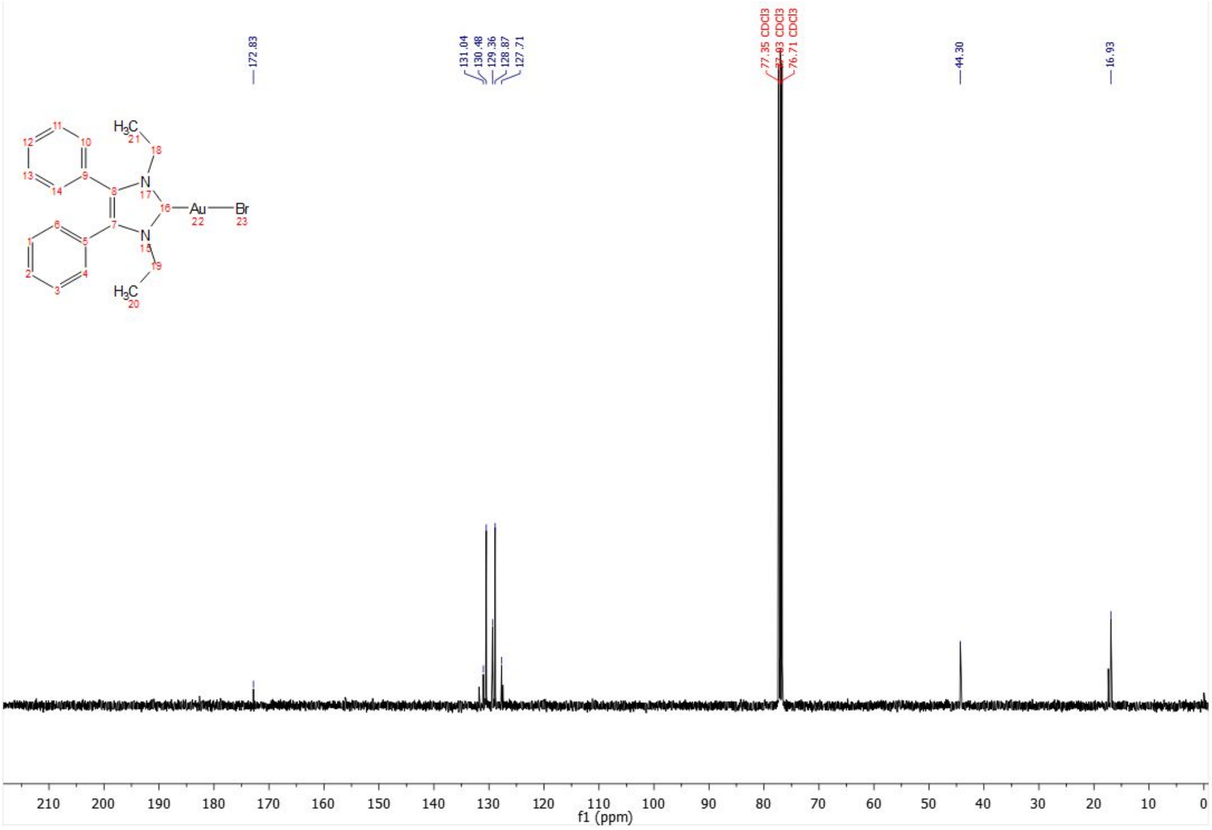

Figure S16. ^{13}C NMR spectrum (101 MHz) of **6** recorded in chloroform-*d*.

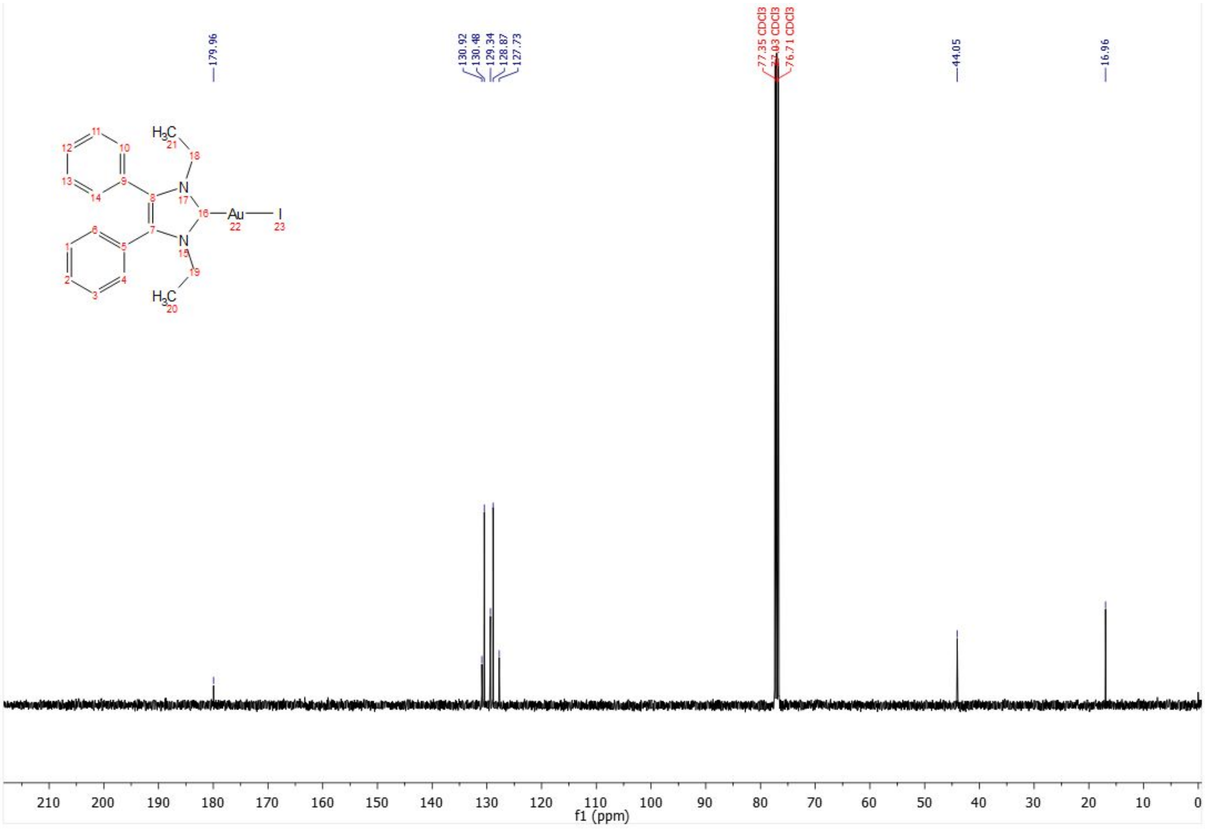

Figure S17. ^{13}C NMR spectrum (101 MHz) of **7** recorded in chloroform-*d*.

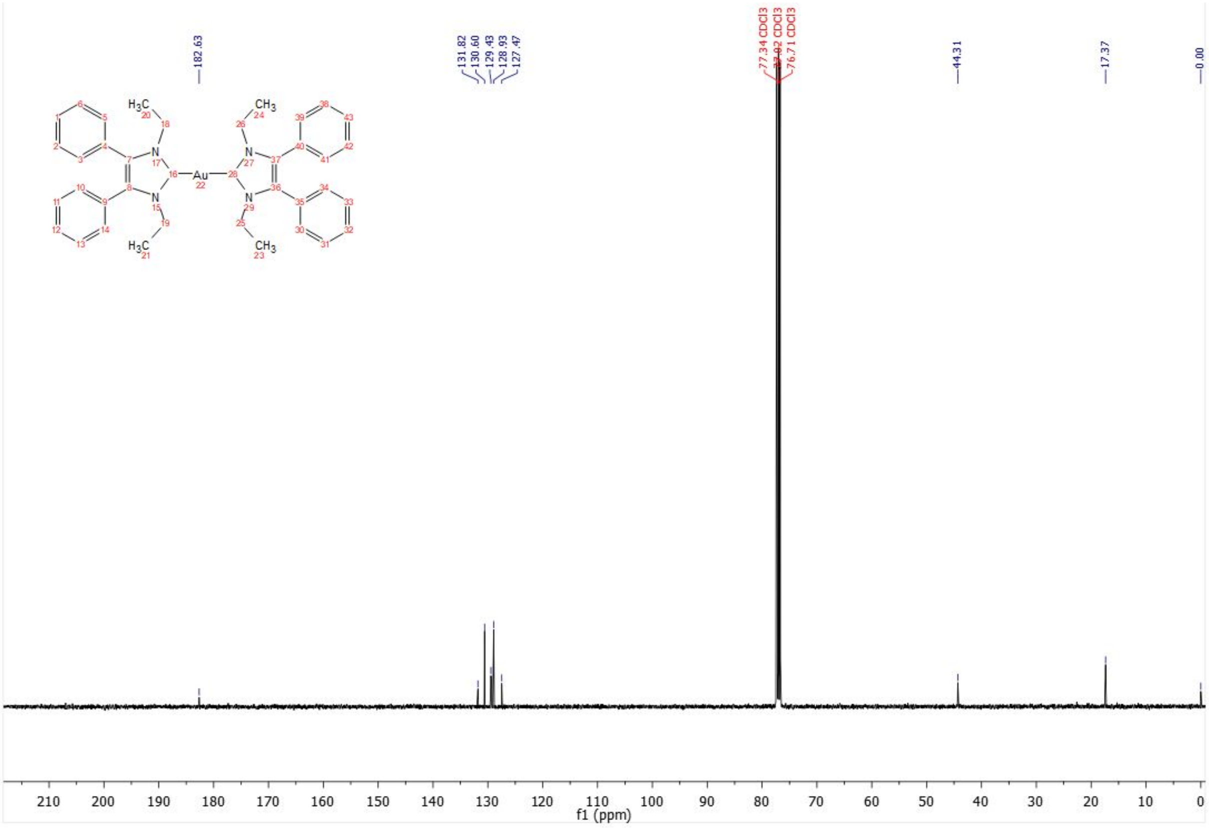

Figure S18. ^{13}C NMR spectrum (101 MHz) of **8** recorded in chloroform-*d*.

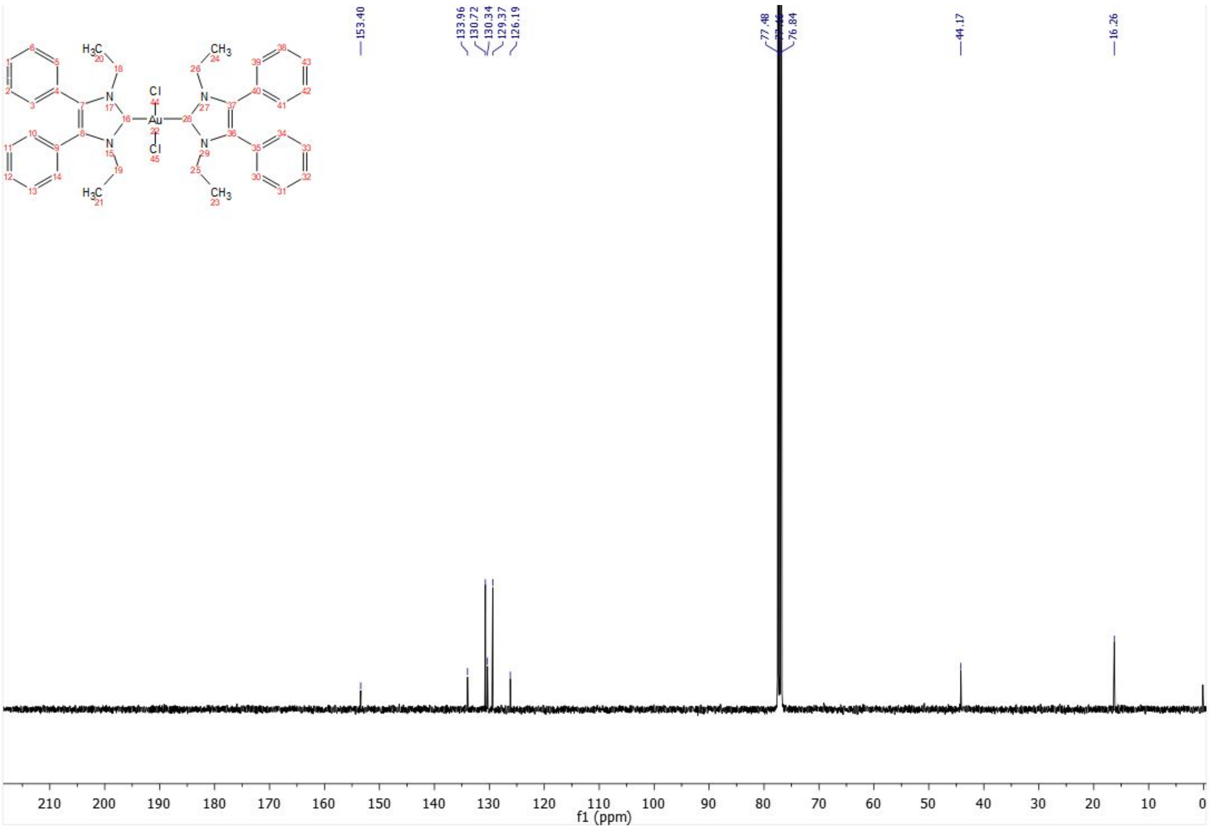

Figure S19. ^{13}C NMR spectrum (101 MHz) of **9** recorded in chloroform-*d*.

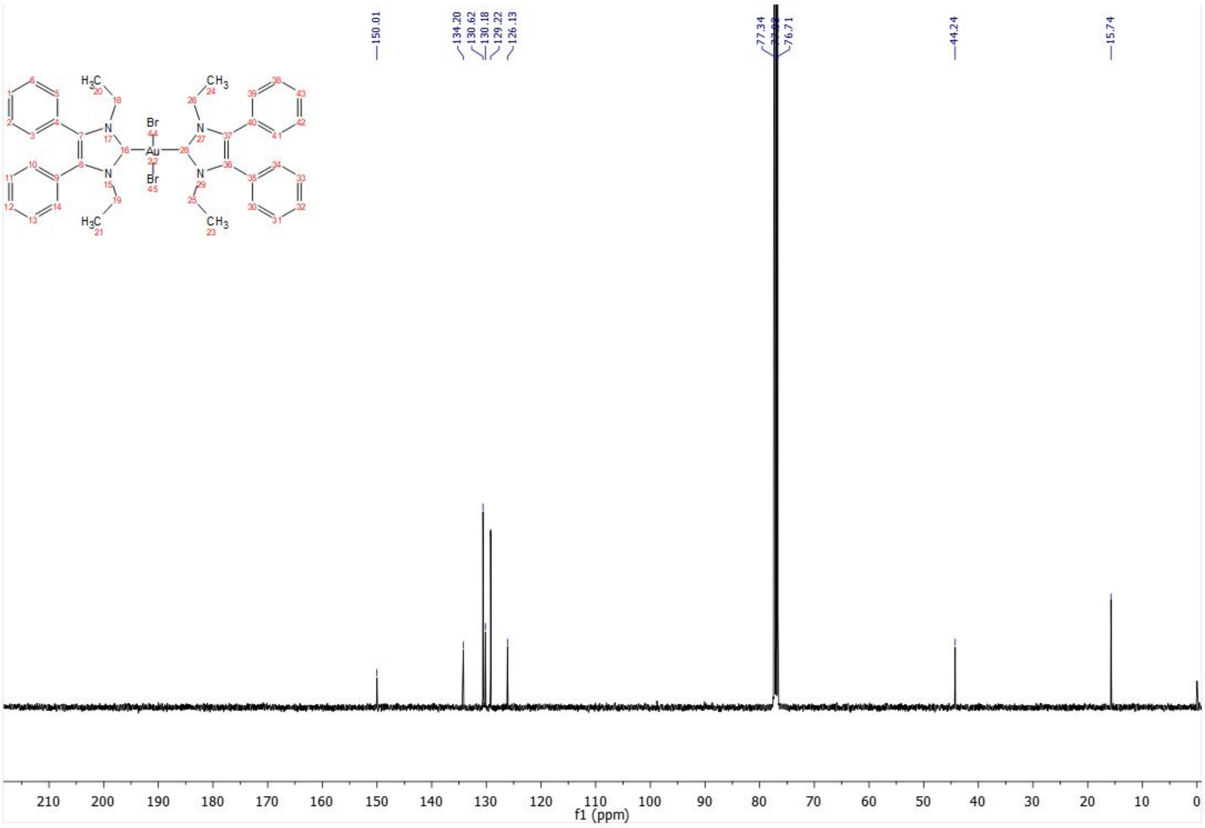

Figure S20. ^{13}C NMR spectrum (101 MHz) of **10** recorded in chloroform-*d*.

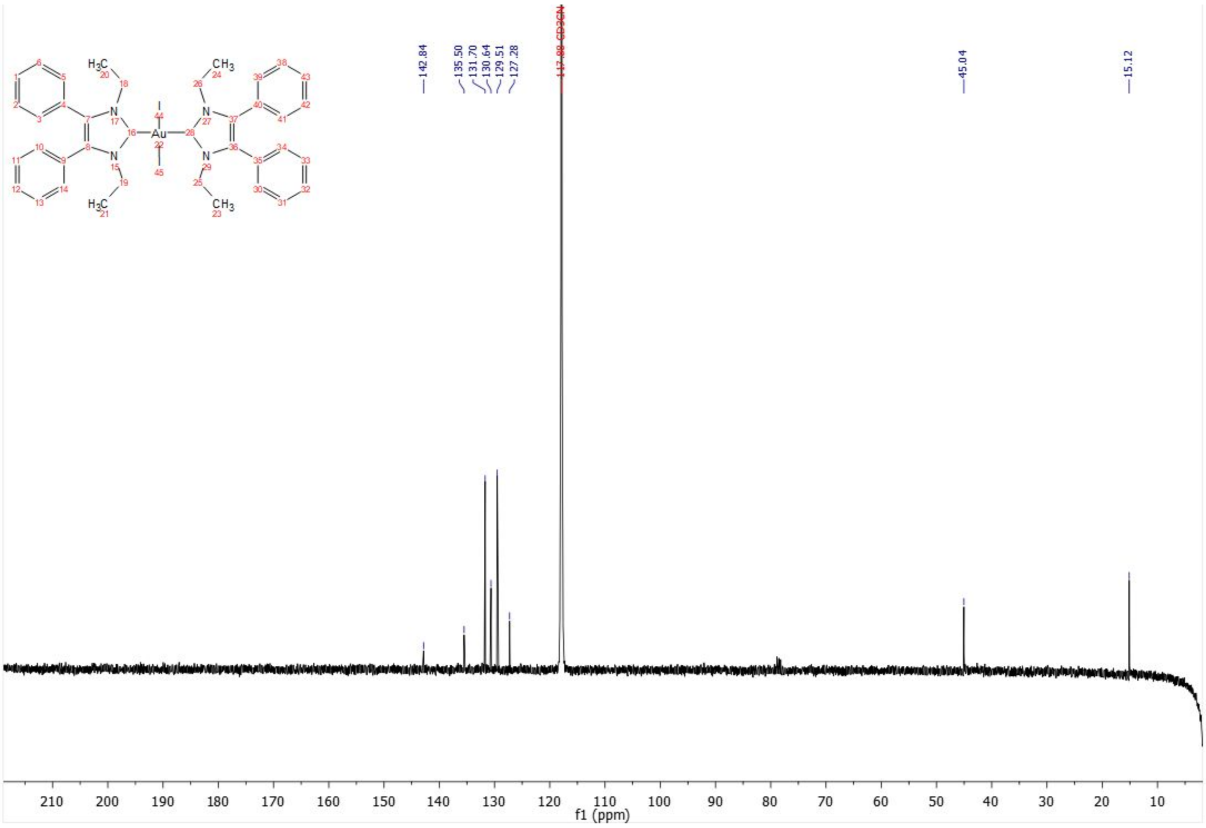

Figure S21. ^{13}C NMR spectrum (101 MHz) of **11** recorded in acetonitrile-d_3.

2. X-ray structures of **6**, **7**, **10**, and **11**

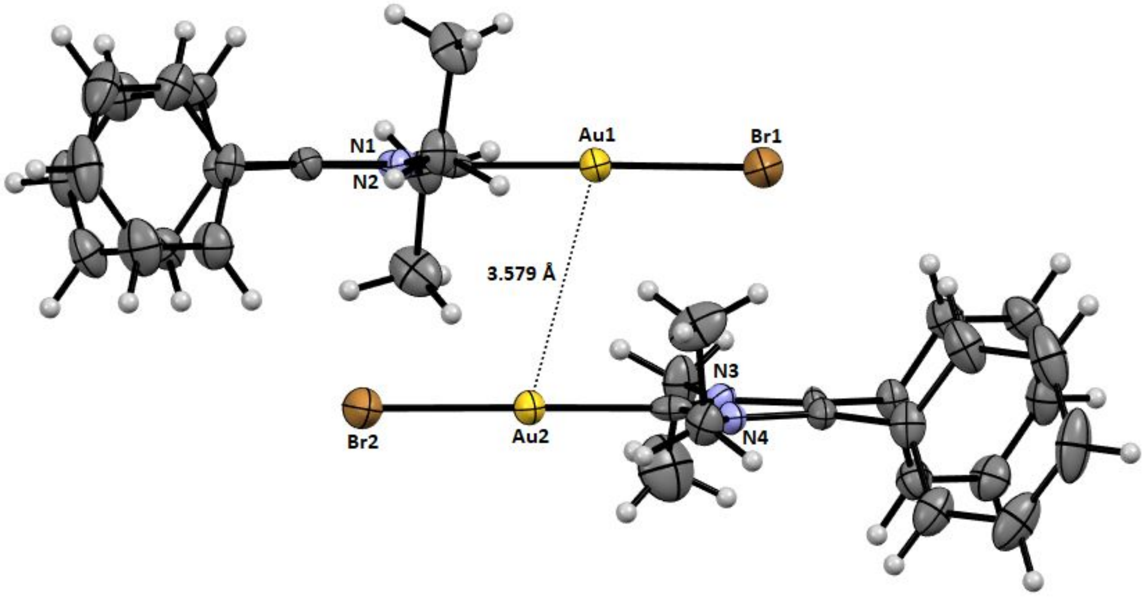

Figure S22. X-ray structure of **6** with Au(I)-Au(I) distance [Å].

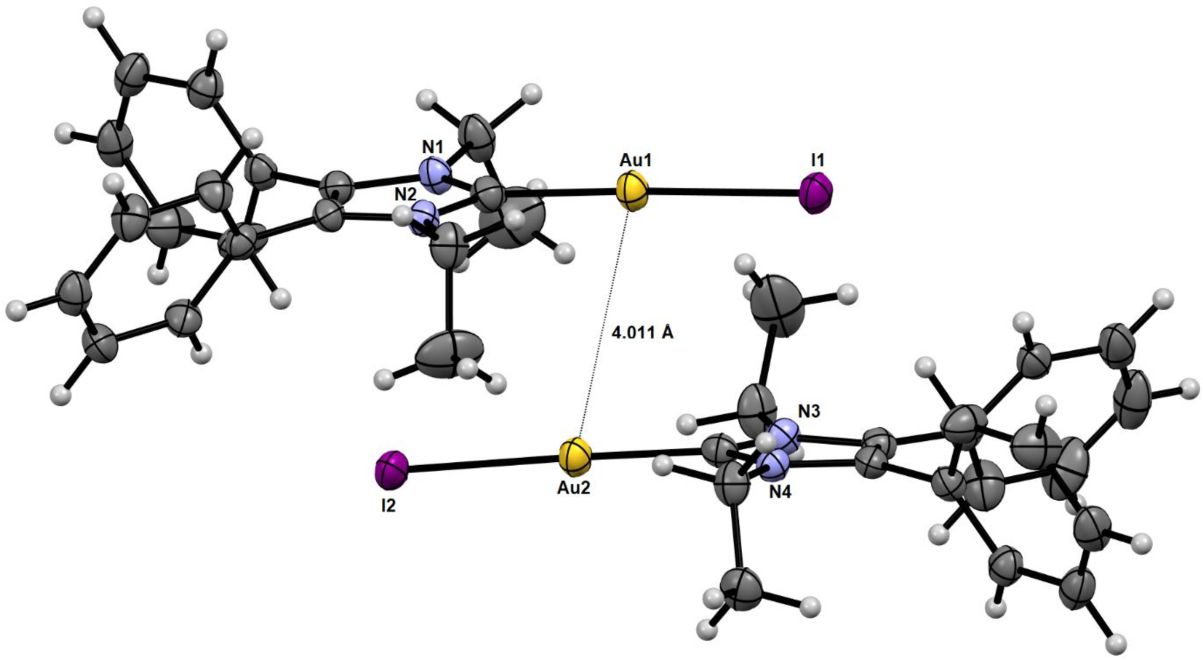

Figure S23. X-ray structure of **7** with Au(I)-Au(I) distance [Å].

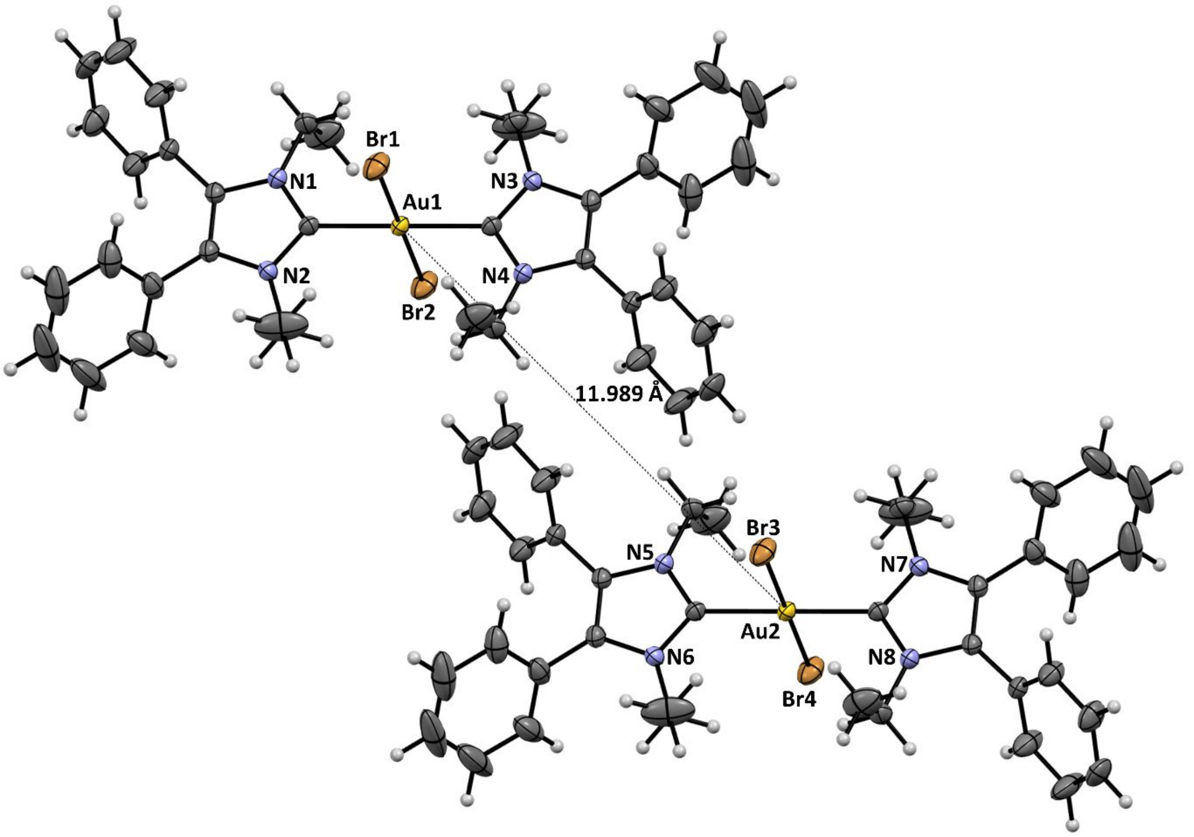

Figure S24. X-ray structure of **10** with Au(I)-Au(I) distance [Å].

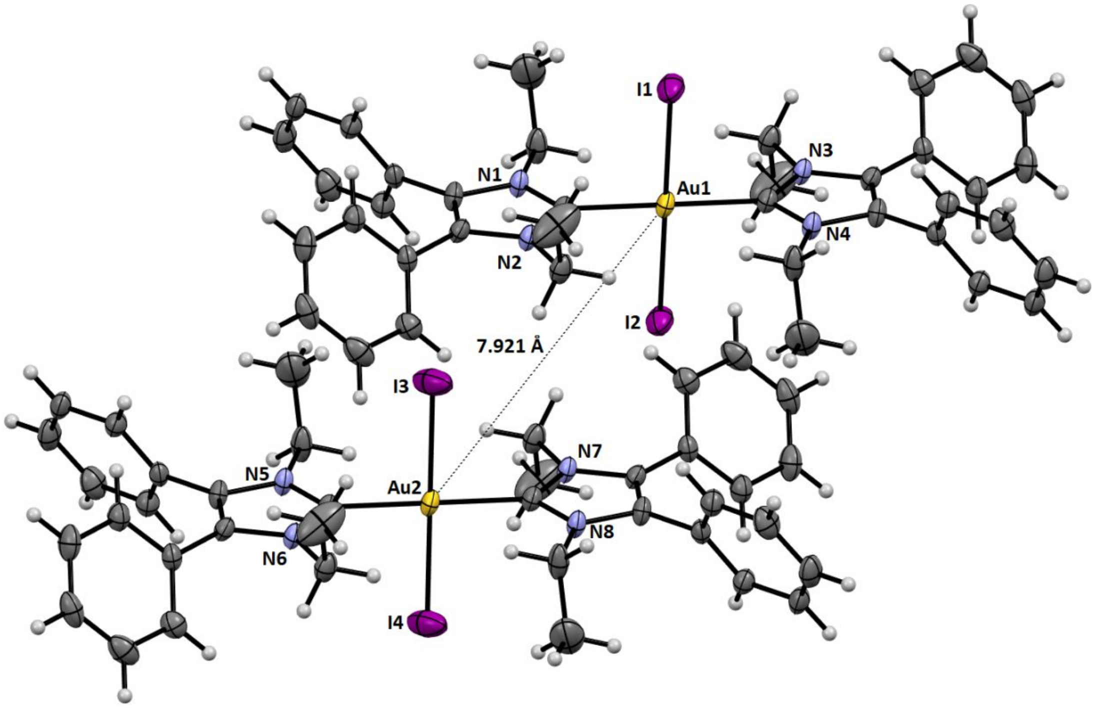

Figure S25. X-ray structure of **11** with Au(I)-Au(I) distance [Å].

3. Crystallographic data and comparison of gold-related crystal parameters of **5-11**

Table S1. Gold-related crystallographic parameters of **5-11** (X = Cl, Br, I).

	Au(I)				Au(III)		
	(NHC)Au(I)X			$[(NHC)Au(I)]^+$	$[(NHC)Au(III)X_2]^+$		
	**5**	**6**	**7**	**8**	**9**	**10**	**11**
Au(I)-halogen [Å]	2.229	2.394	2.584	-	2.227	2.416	2.605
Au(I)-NHC [Å]	1.995	2.028	2.008	2.043	2.049	2.043	2.045
Au(I)-Au(I) [Å]	3.532	3.579	4.011	9.860	8.992	11.898	7.921
NHC-Au(I/III)-halogen/NHC	178.90	179.06	176.84	177.36	175.87	180.00	180.00

[°]							

Table S2. Crystal data and structure refinement for **5**.

Empirical formula	$C_{19}H_{20}AuClN_2$	
Formula weight	508.79	
Temperature	183(2) K	
Wavelength	0.71073 Å	
Crystal system	Triclinic	
Space group	P-1 (no. 2)	
Unit cell dimensions	a = 13.3551(5) Å	α = 94.660(2)°.
	b = 15.2944(7) Å	β = 92.5740(10)°.
	c = 17.8347(8) Å	γ = 92.7260(10)°.
Volume	3622.4(3) $Å^3$	
Z	8	
Density (calculated)	1.866 mg/m^3	
Absorption coefficient	8.270 mm^{-1}	
F(000)	1952	
Crystal size	0.180 x 0.140 x 0.030 mm^3	
Theta range for data collection	1.833 to 27.499°.	
Index ranges	-17<=h<=17, -19<=k<=19, -23<=l<=23	
Reflections collected	82577	
Independent reflections	16616 [R(int) = 0.0304]	
Completeness to theta = 25.242°	99.9 %	
Absorption correction	Semi-empirical from equivalents	
Max. and min. transmission	0.746 and 0.475	
Refinement method	Full-matrix least-squares on F^2	
Data / restraints / parameters	16616 / 0 / 838	
Goodness-of-fit on F^2	1.055	
Final R indices [I>2sigma(I)]	R_1 = 0.0209, wR_2 = 0.0463	
R indices (all data)	R_1 = 0.0269, wR_2 = 0.0482	
Extinction coefficient	0.00100(3)	
Largest diff. peak and hole	1.475 and -0.989 e.$Å^{-3}$	

Table 3. Crystal data and structure refinement for **6**.

Empirical formula	$C_{19}H_{20}AuBrN_2$	
Formula weight	553.25	
Temperature	183(2) K	
Wavelength	0.71073 Å	
Crystal system	Triclinic	
Space group	P-1 (no. 2)	
Unit cell dimensions	a = 13.6224(5) Å	α = 95.2811(13)°.
	b = 15.3368(6) Å	β = 92.6145(12)°.
	c = 17.7056(7) Å	γ = 92.7343(11)°.
Volume	3674.6(2) $Å^3$	
Z	8	
Density (calculated)	2.000 mg/m^3	
Absorption coefficient	10.182 mm^{-1}	
F(000)	2096	
Crystal size	0.250 x 0.170 x 0.090 mm^3	
Theta range for data collection	1.846 to 27.500°.	
Index ranges	-16<=h<=17, -19<=k<=19, -23<=l<=23	
Reflections collected	88334	
Independent reflections	16855 [R(int) = 0.0548]	
Completeness to theta = 25.242°	99.9 %	
Absorption correction	Semi-empirical from equivalents	
Max. and min. transmission	0.333 and 0.137	
Refinement method	Full-matrix least-squares on F^2	
Data / restraints / parameters	16855 / 0 / 838	
Goodness-of-fit on F^2	1.057	
Final R indices [I>2sigma(I)]	R_1 = 0.0349, wR_2 = 0.0975	
R indices (all data)	R_1 = 0.0444, wR_2 = 0.1028	
Extinction coefficient	0.00166(7)	
Largest diff. peak and hole	1.741 and -3.520 e.$Å^{-3}$	

Table S4. Crystal data and structure refinement for **7**.

Empirical formula	$C_{19}H_{20}AuIN_2$	
Formula weight	600.24	
Temperature	183(2) K	
Wavelength	0.71073 Å	
Crystal system	Triclinic	
Space group	P-1 (no. 2)	
Unit cell dimensions	a = 13.7713(6) Å	α = 98.091(2)°.
	b = 16.5520(7) Å	β = 104.240(2)°.
	c = 18.3404(8) Å	γ = 104.275(2)°.
Volume	3835.7(3) $Å^3$	
Z	8	
Density (calculated)	2.079 mg/m^3	
Absorption coefficient	9.281 mm^{-1}	
F(000)	2240	
Crystal size	0.170 x 0.060 x 0.030 mm^3	
Theta range for data collection	1.928 to 27.500°.	
Index ranges	-17<=h<=17, -21<=k<=21, -23<=l<=23	
Reflections collected	125538	
Independent reflections	17597 [R(int) = 0.0318]	
Completeness to theta = 25.242°	99.9 %	
Absorption correction	Semi-empirical from equivalents	
Max. and min. transmission	0.629 and 0.399	
Refinement method	Full-matrix least-squares on F^2	
Data / restraints / parameters	17597 / 0 / 838	
Goodness-of-fit on F^2	1.048	
Final R indices [I>2sigma(I)]	R_1 = 0.0187, wR_2 = 0.0375	
R indices (all data)	R_1 = 0.0250, wR_2 = 0.0390	
Extinction coefficient	0.000598(15)	
Largest diff. peak and hole	2.086 and -1.518 e.$Å^{-3}$	

Table S5. Crystal data and structure refinement for **8**.

Empirical formula	$C_{38}H_{40}AuF_6N_4P$	
Formula weight	894.67	
Temperature	173(2) K	
Wavelength	0.71073 Å	
Crystal system	Tetragonal	
Space group	$P4_32_12$ (no. 96)	
Unit cell dimensions	a = 11.4702(5) Å	α = 90°.
	b = 11.4702(5) Å	β = 90°.
	c = 27.5233(13) Å	γ = 90°.
Volume	3621.1(4) $Å^3$	
Z	4	
Density (calculated)	1.641 mg/m^3	
Absorption coefficient	4.171 mm^{-1}	
F(000)	1776	
Crystal size	0.160 x 0.060 x 0.050 mm^3	
Theta range for data collection	1.923 to 25.727°.	
Index ranges	-13<=h<=13, -13<=k<=13, -33<=l<=33	
Reflections collected	77986	
Independent reflections	3447 [R(int) = 0.0455]	
Completeness to theta = 25.242°	100.0 %	
Absorption correction	Semi-empirical from equivalents	
Max. and min. transmission	0.789 and 0.677	
Refinement method	Full-matrix least-squares on F^2	
Data / restraints / parameters	3447 / 0 / 229	
Goodness-of-fit on F^2	1.108	
Final R indices [I>2sigma(I)]	R_1 = 0.0176, wR_2 = 0.0441	
R indices (all data)	R_1 = 0.0190, wR_2 = 0.0444	
Absolute structure parameter	-0.023(2)	
Extinction coefficient	n/a	
Largest diff. peak and hole	0.823 and -0.640 e.$Å^{-3}$	

Table S6. Crystal data and structure refinement for **9**.

Empirical formula	$C_{38}H_{40}AuCl_2F_6N_4P$	
Formula weight	1050.536	
Temperature	302.00 K	
Wavelength	0.71073 Å	
Crystal system	Orthorhombic	
Space group	Pnma (no. 62)	
Unit cell dimensions	a = 16.4233(8) Å	α = 90°.
	b = 21.0902(9) Å	β = 90°.
	c = 12.4237(5) Å	γ = 90°.
Volume	4303.2(3) $Å^3$	
Z	4	
Density (calculated)	1.622 mg/m^3	
Absorption coefficient	3.763 mm^{-1}	
F(000)	2078.535	
Crystal size	0.16 x 0.14 x 0.06 mm^3	
Theta range for data collection	2.06 to 27.15°.	
Index ranges	-21<=h<=21, -27<=k<=27, -15<=l<=15	
Reflections collected	93283	
Independent reflections	4894 [R(int) = 0.0384]	
Completeness to theta = 25.2417°	99.93 %	
Absorption correction	Semi-empirical from equivalents	
Max. and min. transmission	0.7563 and 0.5659	
Refinement method	Full-matrix least-squares on F^2	
Data / restraints / parameters	4894 / 0 / 259	
Goodness-of-fit on F^2	1.0722	
Final R indices [I>2sigma(I)]	R_1 = 0.0291, wR_2 = 0.0765	
R indices (all data)	R_1 = 0.0342, wR_2 = 0.0827	
Largest diff. peak and hole	2.1356 and -1.8268 e.$Å^{-3}$	

Table S7. Crystal data and structure refinement for **10**.

Empirical formula	$C_{38}H_{40}AuBr_2F_6N_4P$	
Formula weight	1054.49	
Temperature	183.00 K	
Wavelength	0.71073 Å	
Crystal system	Monoclinic	
Space group	$P2_1/c$ (no. 14)	
Unit cell dimensions	a = 8.8616(3) Å	α = 90°.
	b = 18.9516(6) Å	β = 91.6040(10)°.
	c = 11.8956(4) Å	γ = 90°.
Volume	1996.98(11) $Å^3$	
Z	2	
Density (calculated)	1.754 mg/m^3	
Absorption coefficient	5.785 mm^{-1}	
F(000)	1028	
Crystal size	0.11 x 0.08 x 0.02 mm^3	
Theta range for data collection	2.538 to 27.140°.	
Index ranges	-11<=h<=10, -24<=k<=24, -15<=l<=15	
Reflections collected	40798	
Independent reflections	4414 [R(int) = 0.0311]	
Completeness to theta = 25.242°	99.9 %	
Absorption correction	Semi-empirical from equivalents	
Max. and min. transmission	0.8131 and 0.6337	
Refinement method	Full-matrix least-squares on F^2	
Data / restraints / parameters	4414 / 0 / 240	
Goodness-of-fit on F^2	1.062	
Final R indices [I>2sigma(I)]	R_1 = 0.0181, wR_2 = 0.0418	
R indices (all data)	R_1 = 0.0223, wR_2 = 0.0438	
Extinction coefficient	n/a	
Largest diff. peak and hole	1.061 and -0.553 e.$Å^{-3}$	

Table S8. Crystal data and structure refinement for **11**.

Empirical formula	$C_{38}H_{40}AuF_6I_2N_4P$	
Formula weight	1148.47	
Temperature	183.00 K	
Wavelength	0.71073 Å	
Crystal system	Triclinic	
Space group	P-1 (no. 2)	
Unit cell dimensions	a = 7.9209(3) Å	α = 96.8314(15)°.
	b = 10.5213(5) Å	β = 104.0017(15)°.
	c = 12.6129(5) Å	γ = 95.7547(15)°.
Volume	1003.54(7) Å^3	
Z	1	
Density (calculated)	1.900 mg/m^3	
Absorption coefficient	5.303 mm^{-1}	
F(000)	550	
Crystal size	0.12 x 0.04 x 0.015 mm^3	
Theta range for data collection	2.674 to 27.138°.	
Index ranges	-10<=h<=10, -13<=k<=13, -16<=l<=16	
Reflections collected	20031	
Independent reflections	4427 [R(int) = 0.0462]	
Completeness to theta = 25.242°	99.6 %	
Absorption correction	Semi-empirical from equivalents	
Max. and min. transmission	0.7673 and 0.6492	
Refinement method	Full-matrix least-squares on F^2	
Data / restraints / parameters	4427 / 0 / 216	
Goodness-of-fit on F^2	1.063	
Final R indices [I>2sigma(I)]	R_1 = 0.0277, wR_2 = 0.0607	
R indices (all data)	R_1 = 0.0327, wR_2 = 0.0638	
Extinction coefficient	n/a	
Largest diff. peak and hole	0.678 and -1.165 e.Å^{-3}	

4. Reaction of **7** with non-thiol containing amino acids

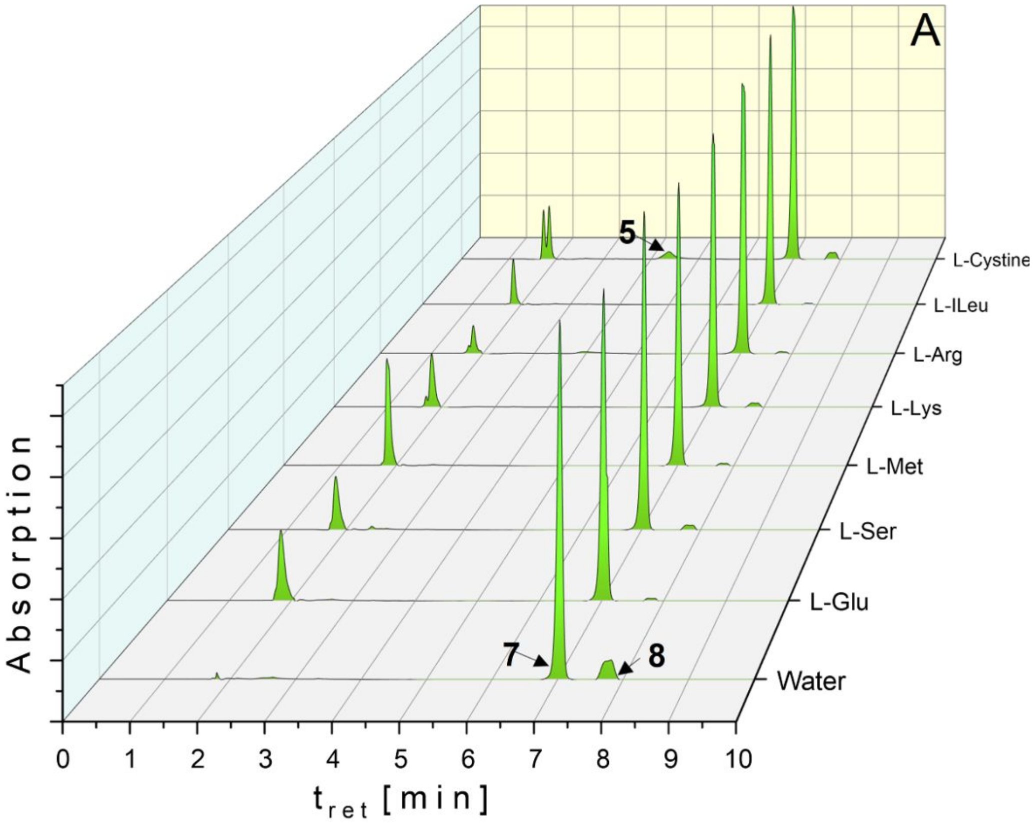

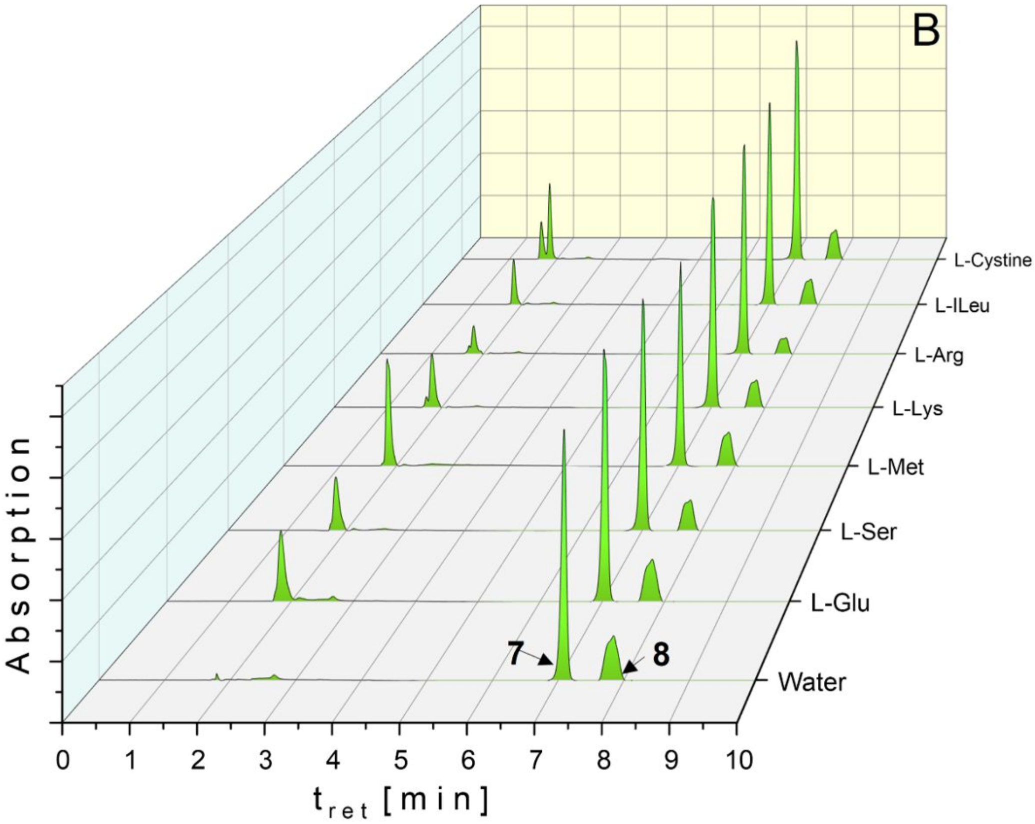

Figure S 26. Formation of $[(NHC)_2Au(I)]^+$ (**8**) from **7** in presents of 20 eq. non-thiol containing amino acid in ACN/water = 50/50 (v/v) at t_{0h} (A) and t_{24h} (B).

5. HPLC chromatograms of **7** with 20 eq. GSH

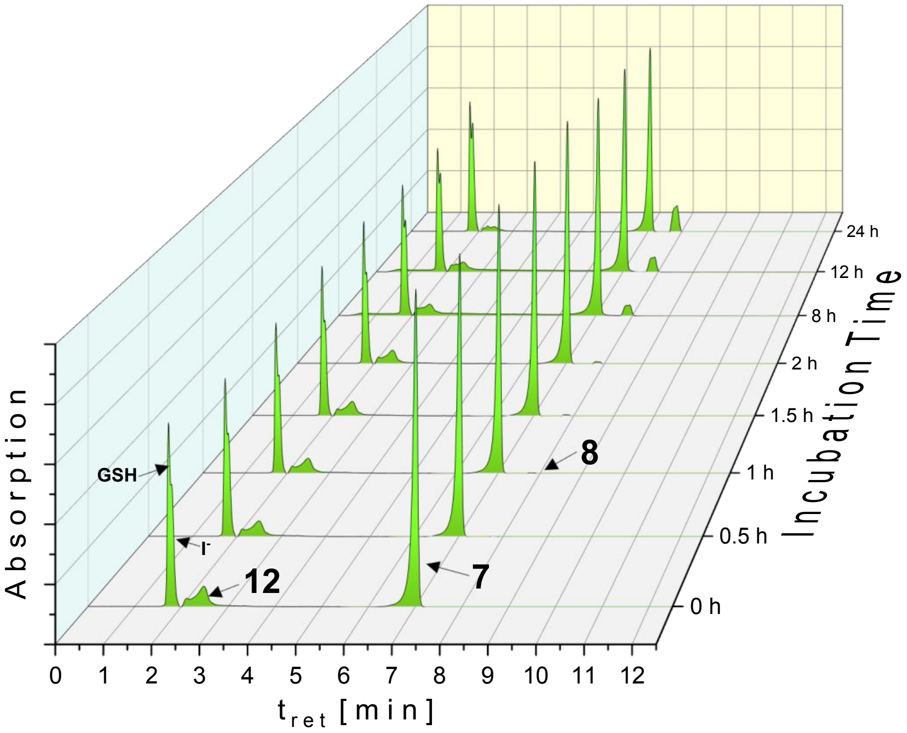

Figure S27. HPLC-chromatograms of **7** incubated with 20 eq. GSH in ACN/water = 50/50 (v/v) up to 24 h.

6. HPLC chromatograms of **5** with 20 eq. of NADPH or ascorbate

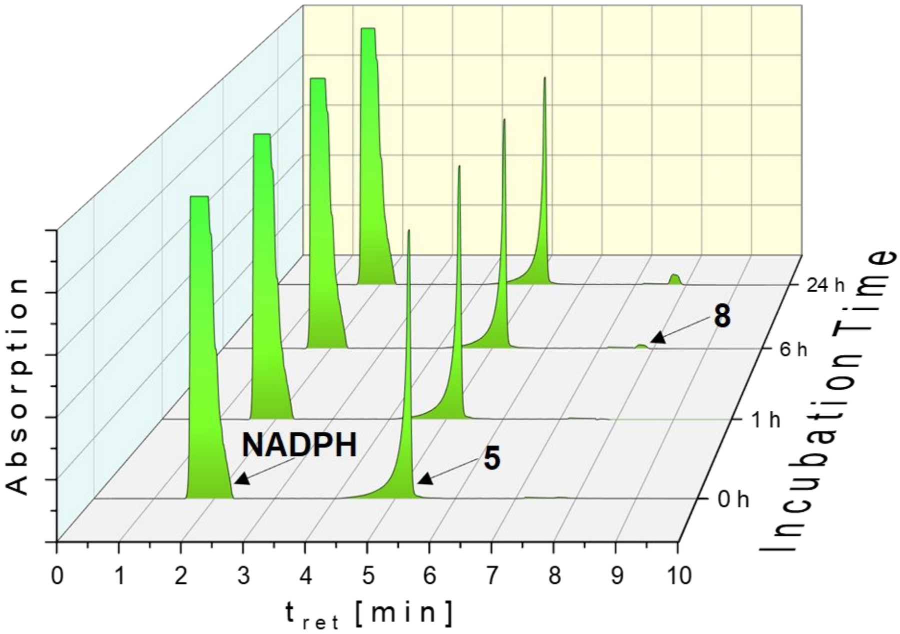

Figure S28. HPLC chromatograms of **5** (solution: ACN/water = 50/50 (v/v)) in presence of 20 eq. NADPH. Complex concentration 0.5 mM.

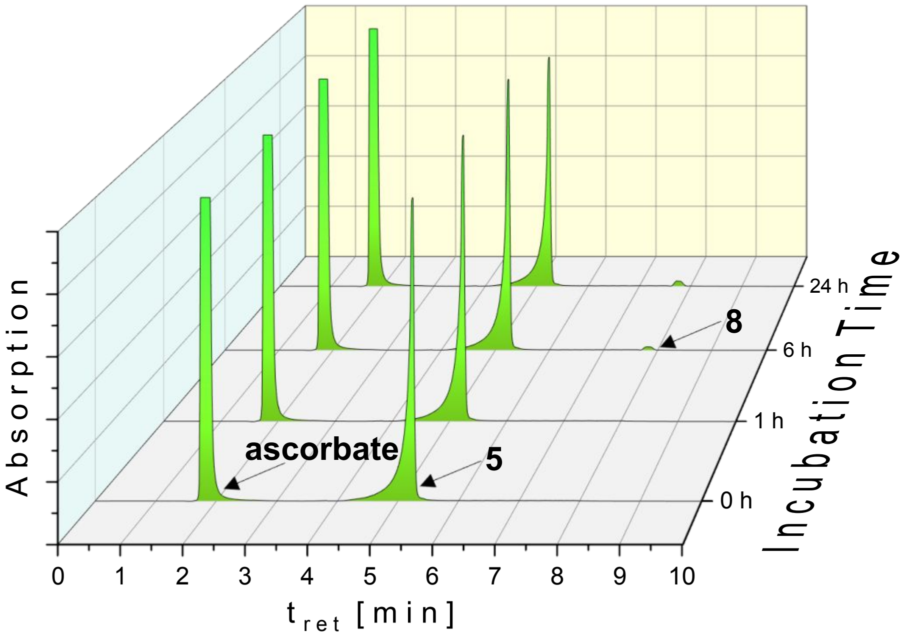

Figure S29. HPLC chromatograms of **5** (solution: ACN/water = 50/50 (v/v)) in presence of 20 eq. sodium ascorbate. Complex concentration 0.5 mM.

7. HR-MS experiments with **5** or **11** and GSH

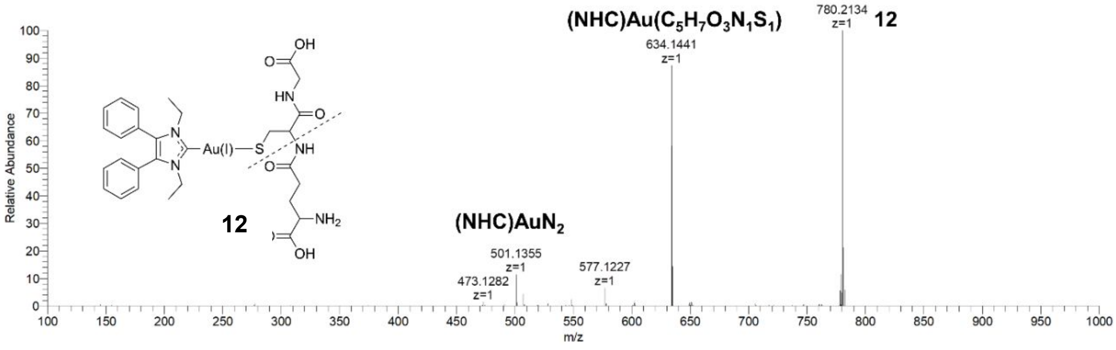

Figure S30. HCD fragmentation spectrum of m/z 780 (z = 1) corresponding to (NHC)Au(I)-GSH (**12**). Adduct formation with the collision gas N_2 has also been observed for similar gold(I) complexes.[14]

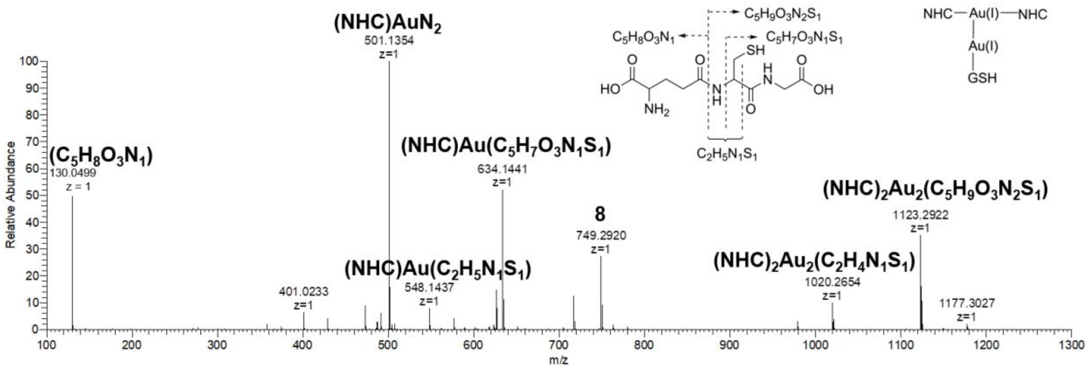

Figure S31. HCD fragmentation spectrum of m/z 626 (z = 2) corresponding to $(NHC)_2Au_2$-GSH.

Table S9. Experimental and calculated *m/z* values for all identified ions.

Species	m $_{exp.}$	m $_{calc.}$	Error [ppm]
$[C_5H_8O_3N_1]^+$	130.0449	130.0449	0.00
$[GSH + H]^+$	308.0917	308.0911	1.95
$[(NHC)AuN_2]^+$	501.1355	501.1348	1.40
$[(NHC)Au(C_2H_5N_1S_1)]^+$	548.1437	548.1429	1.46
$[(NHC)Au(C_5H_7O_3N_1S_1)]^+$	634.1441	634.1433	1.26
$[(NHC)_2Au]^+$ (**8**)	749.2930	749.2913	2.27
$[(NHC)AuGSH]^+$ (**12**)	780.2143	780.2125	2.31
$[(NHC)_2Au_2(C_2H_4N_1S_1)]^+$	1020.2654	1020.2643	1.08
$[(NHC)_2Au_2(C_5H_9O_3N_2S_1)]^+$	1123.2921	1123.2912	0.80
$[(NHC)_2Au_2GS^-]^+$	1252.3367	1252.3338	2.32

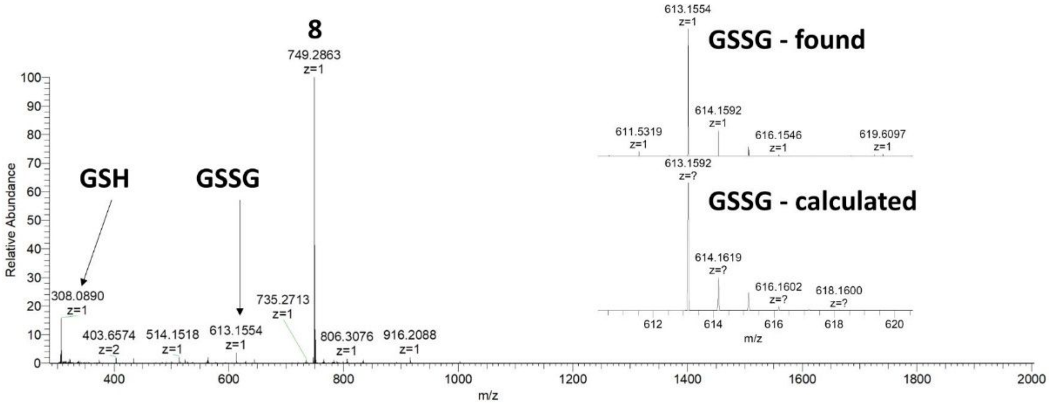

Figure S32. Full mass spectrum obtained from a 50/50 mixture of **11** in ACN and GSH in water after 1.5 min.

8. HPLC chromatograms of 5 with 20 eq. GSH in PBS

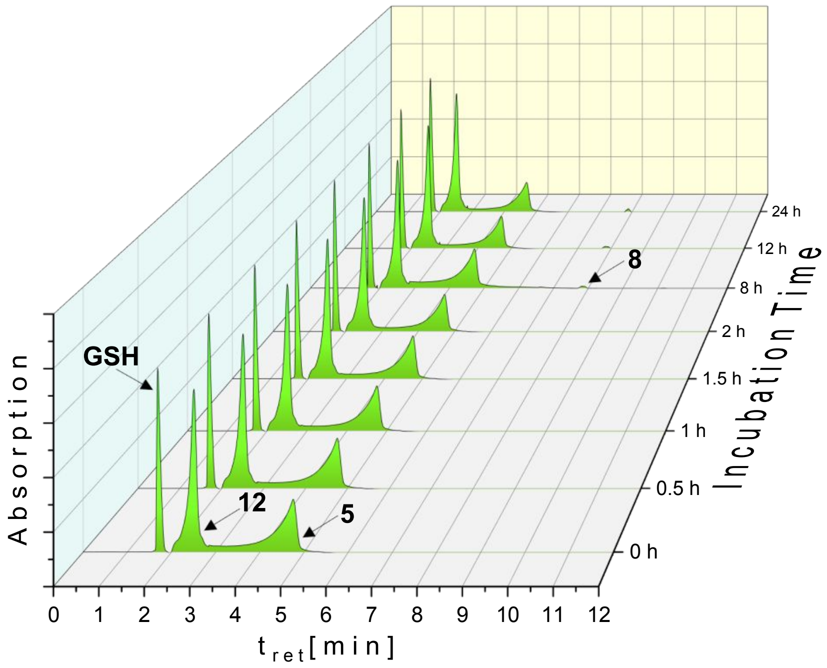

Figure S33. HPLC-chromatograms of **5** incubated with 20 eq. GSH in ACN/PBS = 50/50 (v/v) up to 24 h.

9. HPLC chromatograms of the conversation from **11** to **8** in the presence of RPMI 1640

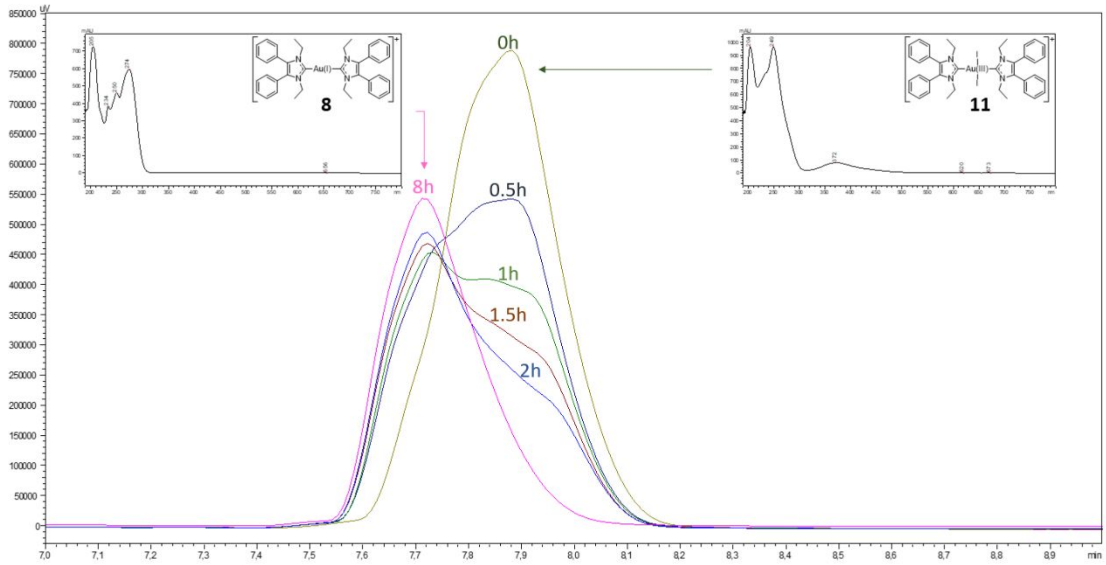

Figure S34. HPLC chromatograms of time dependent conversion from **11** to **8** in ACN/RPMI 1640 = 50/50 (v/v).
